# Titanium mesh and tray systems for mandibular continuity reconstruction with bone grafting: a systematic review and proportional meta-analysis of device-related complications

**DOI:** 10.3389/fbioe.2026.1915266

**Published:** 2026-08-26

**Authors:** Tingting Hou, Xinrui Yin, Kai Lu

**Affiliations:** 1 Department of Oral and Maxillofacial Surgery, Dandong Central Hospital, Dandong, Liaoning, China; 2 Department of Anesthesiology, Aerospace Center Hospital, Beijing, China

**Keywords:** bone grafting, device-related complications, mandibular continuity defects, mandibular reconstruction, proportional meta-analysis, systematic review, titanium mesh, titanium tray

## Abstract

**Background:**

Titanium mesh/tray systems have been used with bone grafting for mandibular continuity reconstruction, but evidence is scattered across small cohorts and device eras. We systematically reviewed this literature and quantified device-related complications while accounting for cohort overlap and outcome-specific denominators.

**Methods:**

PubMed, Embase, Web of Science, Scopus, the Cochrane Central Register of Controlled Trials (CENTRAL), and ClinicalTrials.gov were searched for human clinical studies evaluating titanium mesh/tray systems used as graft-containing reconstruction chambers for segmental or continuity mandibular defects. Representative independent cohorts were selected to avoid double-counting. The primary outcomes were device exposure, infection, mechanical failure, and unplanned reoperation or device removal. Pooled proportions were estimated using logistic-normal generalized linear mixed models. Risk of bias and certainty of evidence were assessed using JBI, ROBINS-I, and GRADE.

**Results:**

After screening 736 unique records and assessing 53 full-text reports, 40 reports were retained for quantitative synthesis, qualitative interpretation, evidence mapping, or cohort adjudication. Ten representative independent cohorts were included, of which eight contributed to at least one primary pooled outcome. In the primary analyses, the pooled proportions were 8.8% (95% CI, 3.7%–19.4%) for device exposure, 14.5% (95% CI, 8.4%–23.8%) for infection, 5.3% (95% CI, 2.0%–13.2%) for mechanical failure, and 14.5% (95% CI, 8.0%–24.9%) for unplanned reoperation or device removal. Sensitivity analyses incorporating one cohort without confirmable minimum follow-up, and excluding the single cohort reported using reconstruction-site-level denominators, were directionally consistent. Graft consolidation or mandibular continuity restoration, implant placement, and prosthetic rehabilitation were summarized as exploratory downstream outcomes. Only two cohorts used additively manufactured patient-specific systems, precluding reliable subgroup inference. Certainty of evidence was very low for all primary outcomes.

**Conclusion:**

Titanium mesh/tray systems may be considered in selected clinical contexts for mandibular continuity reconstruction, but exposure, infection, and unplanned reoperation or device removal remain important concerns. Current estimates should be interpreted as exploratory clinical benchmarks rather than definitive evidence of safety or effectiveness. Prospective multi-institutional studies with standardized outcome definitions and appropriate comparator groups are needed.

## 1 Introduction

Mandibular continuity defects may result from tumour resection, trauma, infection, osteoradionecrosis, congenital or developmental conditions, or failed previous reconstruction (Ruf et al., 2025; Brown et al., 2017). These defects can compromise facial contour, occlusal stability, mastication, speech, and subsequent oral rehabilitation (Ruf et al., 2025; Brown et al., 2017). Vascularized bone flaps remain a major reconstructive option, particularly for extensive or complex defects. In selected clinical settings, however, titanium mesh/tray systems combined with bone grafting have also been used, with the titanium device serving as the primary reconstructive framework for graft containment, contour restoration, and mechanical stabilization (Brown et al., 2017; Ikawa et al., 2016; Ripamonti and Petit, 2009; Iino et al., 2009; Yamada et al., 2016).

Titanium mesh/tray systems include preformed, stock, manually adapted, model-bent, cast, CAD/CAM-assisted, and additively manufactured patient-specific designs (Ikawa et al., 2016; Ripamonti and Petit, 2009; Iino et al., 2009; Hodgdon et al., 2018; Hoefert et al., 2026; Zhao et al., 2023; Onodera et al., 2023). Despite differences in manufacturing and customization, these systems share the functional concept of a graft-containing reconstruction chamber that maintains space, contains particulate or block graft material, and supports mandibular continuity restoration during healing (Ripamonti and Petit, 2009; Iino et al., 2009; Hodgdon et al., 2018; Onodera et al., 2023). Advances in virtual surgical planning, three-dimensional modelling, and additive manufacturing have increased interest in patient-specific systems, but the clinical evidence remains concentrated in small case series, technical reports, and institution-specific cohorts (Hodgdon et al., 2018; Hoefert et al., 2026; Zhao et al., 2023; Onodera et al., 2023; Huang et al., 2023; Ostrovidov et al., 2023).

The device-related complication profile of titanium mesh/tray systems has not been systematically quantified. Reported outcomes include device exposure, infection, mechanical failure, unplanned reoperation or device removal, graft consolidation, dental implant placement, and prosthetic rehabilitation (Ripamonti and Petit, 2009; Iino et al., 2009; Hodgdon et al., 2018; Onodera et al., 2023; Wang et al., 2021; DeGould and Goldberg, 1991; Zhou et al., 2010). Interpretation is limited by small sample sizes, heterogeneous indications and grafting protocols, variable follow-up, and potential overlap among reports from the same institutions or device eras (Ripamonti and Petit, 2009; Iino et al., 2009; Yamada et al., 2016; Onodera et al., 2023; Huang et al., 2023; Ostrovidov et al., 2023; Zhou et al., 2010; Miyamoto et al., 2014; Farid Shehab et al., 2018). Consequently, pooled estimates of device-related complications and the influence of manufacturing approach and denominator decisions remain uncertain.

We therefore conducted a systematic review and proportional meta-analysis of titanium mesh/tray systems used with bone grafting for mandibular continuity reconstruction. The primary objective was to estimate pooled proportions of device exposure, infection, mechanical failure, and unplanned reoperation or device removal. Secondary and exploratory objectives were to summarize graft consolidation or continuity restoration, dental implant placement, prosthetic rehabilitation, manufacturing approach, and certainty of evidence while minimizing double-counting through cohort-level adjudication.

## 2 Methods

### 2.1 Protocol and registration

This systematic review and proportional meta-analysis was conducted and reported in accordance with the Preferred Reporting Items for Systematic Reviews and Meta-Analyses (PRISMA) 2020 statement (Page et al., 2021). The review protocol was prospectively registered in the International Prospective Register of Systematic Reviews (PROSPERO; CRD420261428392) on 19 June 2026, before the formal literature searches and subsequent study selection, data extraction, cohort adjudication, and statistical analysis (Booth et al., 2012). The review evaluated clinical outcomes and device-related complications of titanium mesh/tray systems used with bone grafting for mandibular continuity reconstruction. As this study used previously published data, institutional ethics approval and informed consent were not required.

### 2.2 Eligibility criteria

Studies were eligible if they reported human clinical data on titanium mesh/tray systems used with bone grafting or osteogenic adjuncts for reconstruction of segmental or continuity mandibular defects. For this review, titanium mesh/tray systems was used as the umbrella term for mesh-, tray-, or cage-type titanium constructs that functioned as graft-containing reconstruction chambers. Eligible defects included those resulting from benign or malignant tumour resection, trauma, infection, osteoradionecrosis, congenital or developmental conditions, or failed previous reconstruction. For patients with failed previous reconstruction, eligibility was determined according to the index reconstruction evaluated in the study: cases were eligible when the current reconstruction used a titanium mesh/tray system with bone grafting, irrespective of the previous reconstructive method. Both immediate and delayed reconstructions were eligible.

These clinical settings were not assumed to be interchangeable. Eligibility was based on the shared presence of a mandibular continuity defect treated using the same device-supported grafting principle, while clinically relevant differences in defect etiology and extent, reconstruction timing, radiotherapy, soft-tissue reconstruction, graft type, and adjunctive procedures were extracted for structured interpretation.

The intervention of interest was a titanium mesh/tray system functioning as a graft-containing, space-maintaining, contour-restoring, or load-sharing reconstruction chamber. Custom-made, CAD/CAM-assisted, additively manufactured patient-specific, preformed, stock, model-bent, cast, and manually adapted systems were eligible when they supported mandibular continuity reconstruction with bone grafting or other osteogenic adjuncts. A comparator group was not required.

We excluded studies limited to localized alveolar ridge augmentation, sinus floor elevation, minor peri-implant bone augmentation, fracture fixation, ordinary reconstruction plates without a graft-containing chamber function, vascularized bone flap-dominant reconstruction as the index procedure, non-titanium scaffold or tray systems, and animal, *in vitro*, or finite-element-only studies. Solid patient-specific implants and alloplastic porous titanium prostheses used primarily as direct segmental bone substitutes without a graft-containing chamber function were excluded from the primary synthesis.

Case series, cohort studies, comparative clinical studies, and case reports were eligible for qualitative assessment if they reported at least one relevant clinical, radiographic, reconstructive, or device-related outcome. The primary proportional meta-analysis included only independent clinical cohorts with extractable event counts and denominators for eligible segmental or continuity mandibular reconstructions. Cohorts were required to include at least three eligible patients and to report a minimum follow-up of six months. A cohort meeting all other eligibility criteria but lacking sufficient follow-up information to confirm the six-month threshold was retained in the systematic review and contribution mapping but excluded from the primary pooled estimates and included only in sensitivity analyses. Case reports, technical reports, overlapping reports, cohorts with fewer than three eligible patients, and studies with non-separable eligible denominators or outcome events were retained for qualitative synthesis or evidence mapping when relevant but were not included in the primary pooled estimates.

### 2.3 Information sources and search strategy

The formal literature searches were conducted on 20 June 2026 in PubMed, Embase, Web of Science, Scopus, the Cochrane Central Register of Controlled Trials, and ClinicalTrials.gov, covering each source from inception through that date. No restrictions were applied regarding publication year, language, or country. The search strategy combined terms related to mandibular reconstruction, segmental or continuity mandibular defects, titanium mesh/tray systems, custom-made or preformed devices, computer-assisted design, additive manufacturing, bone grafting, and mandibular continuity restoration (Rethlefsen et al., 2021). The complete search strategies, including search fields, syntax, and source-specific adaptations, are provided in Supplementary Table S1.

The reference lists of eligible studies and relevant review articles were also screened to identify additional potentially eligible records (Thomas et al., 2019). Records identified through reference-list screening, citation checking, or full-text retrieval were assessed using the same eligibility criteria as records identified through the formal searches.

### 2.4 Study selection and full-text assessment

After de-duplication, two reviewers independently screened titles and abstracts against the predefined eligibility criteria (Thomas et al., 2019). Records were excluded at this stage if they were clearly unrelated to mandibular continuity reconstruction, did not evaluate titanium mesh/tray systems, were limited to non-clinical research, or addressed localized alveolar or peri-implant bone augmentation. Records considered potentially eligible, or for which eligibility could not be determined from the title and abstract, were retrieved for full-text assessment. Inter-reviewer agreement at the title-and-abstract stage was substantial (Cohen’s κ = 0.83) (May 1994).

The same two reviewers independently assessed the full texts, with disagreements resolved through discussion and, when necessary, consultation with a third reviewer. Reports were assigned to one of three evidence categories according to cohort independence, clinical relevance, and outcome extractability. Independent clinical cohorts with eligible segmental or continuity mandibular reconstructions and extractable event counts and denominators were considered for the primary proportional meta-analysis. Case reports, technical reports, overlapping reports, and studies with non-separable eligible subgroup denominators or outcome events were retained for qualitative synthesis or evidence mapping when relevant. Contextual studies, including reports of alloplastic porous titanium prostheses and historical studies that informed the broader reconstructive landscape but did not meet the primary synthesis criteria, were summarized narratively where appropriate.

Overlapping or partially overlapping patient populations were resolved through a predefined cohort-adjudication strategy, in which related reports were reviewed jointly and a single representative cohort was selected for quantitative synthesis before denominator rules were applied.

### 2.5 Management of overlapping cohorts and denominator decisions

Potentially overlapping cohorts were assessed by comparing study institution, author group, recruitment period, device type, reconstructive approach, sample size, and, when available, patient-level characteristics. When multiple reports were judged to describe the same or partially overlapping patient population, a single representative cohort was selected for quantitative synthesis based on eligibility, completeness of outcome reporting, and clarity of the eligible denominator. Sample size and follow-up duration were considered after eligibility and outcome completeness. Earlier reports, case reports, or subgroup publications from the same cohort were retained only as supplementary sources for qualitative interpretation, evidence mapping, or clarification of cohort characteristics and were not counted again in the quantitative denominators.

For each representative cohort, the eligible denominator was defined as the number of segmental or continuity mandibular reconstructions performed using a titanium mesh/tray system with bone grafting. In mixed cohorts, subgroup denominators and events were included only when they could be assigned to eligible reconstructions without ambiguity. Marginal defects, localized alveolar procedures, non-titanium systems, vascularized bone flap-dominant index reconstructions, and cases in which the titanium device did not function as a graft-containing reconstruction chamber were excluded when separable. Studies with non-separable eligible denominators or outcome events were retained for qualitative synthesis or evidence mapping but were not included in the primary proportional meta-analysis.

Outcome-specific denominator units were recorded as reported, including patient-level and reconstruction-site-level units. Patient-level conversion was not undertaken when outcome-specific patient-level event counts could not be established unambiguously. The influence of the cohort reporting reconstruction-site-level denominators was evaluated in an additional sensitivity analysis. Planned or protocol-based device removal, staged removal for dental implant placement, elective or cosmetic removal, and tumour recurrence-related removal were not counted as device-related unplanned reoperation or device removal. When exposure and infection were reported only as a combined event category, the event was not entered into both separate outcome pools. Cohort-specific overlap adjudication, eligible denominators, denominator units, and analytic contributions are summarized in Supplementary Table S2.

### 2.6 Data extraction and outcome definitions

Data were extracted independently by two reviewers using a standardized extraction form (Lasserson et al., 2019). Disagreements were resolved through discussion and, when necessary, consultation with a third reviewer. Extracted study-level variables included first author, publication year, country, institution, study design, recruitment period, cohort size, eligible denominator, follow-up duration, defect etiology, defect type, location and extent, reconstruction timing, radiotherapy status, soft-tissue reconstruction or coverage, titanium device type, manufacturing or shaping method, graft type, osteogenic or biologic adjuncts, and reported clinical, radiographic, reconstructive, and device-related outcomes. For each outcome, the event count, denominator, denominator unit, and source of extraction were recorded. Denominators were recorded separately when outcomes were reported by patient, mandibular defect, or reconstruction site.

The primary device-related outcomes were device exposure, infection, mechanical failure, and unplanned reoperation or device removal. Device exposure was defined, according to the original report, as intraoral or extraoral exposure of any component of the titanium mesh/tray system. Infection included graft-site, device-related, or reconstruction-site infection as reported by the study. When exposure and infection were reported only as a combined category, the event was not assigned to both separate outcome pools. Mechanical failure included fracture, breakage, deformation, loosening, collapse, or other structural failure of the titanium mesh/tray system. Because few mechanical failures were observed, this outcome was treated as a sparse-event estimate requiring cautious interpretation.

Unplanned reoperation or device removal was defined as an unplanned secondary intervention related to a device, graft, wound, or reconstruction complication. Planned or protocol-based removal, staged removal for dental implant placement, elective or cosmetic removal, and removal attributable primarily to tumour recurrence were excluded. When revision surgery and device removal represented the same clinical episode, the event was counted once under the combined outcome of unplanned reoperation or device removal.

Exploratory outcomes included graft consolidation or restoration of mandibular continuity, dental implant placement, prosthetic rehabilitation, functional or occlusal outcomes, mouth opening, mastication or diet, patient-reported outcomes, and aesthetic or contour-related outcomes. Because definitions of graft consolidation and continuity restoration varied across studies, these outcomes were not treated as primary pooled outcomes. Radiographic bony continuity or graft consolidation was prioritized when available, followed by clinically reported continuity or reconstructive success. Histological bone formation, volumetric graft retention, and descriptive reports of success were recorded separately and were not assumed to represent equivalent outcomes.

When a study reported multiple definitions or follow-up time points for the same outcome, the definition most closely aligned with the review outcome and the longest clinically relevant follow-up were extracted. Outcomes for which event counts or denominators could not be assigned unambiguously to the eligible subgroup were classified as non-separable and excluded from the primary proportional meta-analysis.

### 2.7 Risk of bias and certainty of evidence

Risk of bias was assessed independently by two reviewers, with disagreements resolved through discussion and, when necessary, consultation with a third reviewer. Single-arm case series and cohort-like case series were assessed using the Joanna Briggs Institute Critical Appraisal Checklist for Case Series (Munn et al., 2020; Munn et al., 2023). Case reports retained for qualitative synthesis or evidence mapping were assessed, when applicable, using the Joanna Briggs Institute Critical Appraisal Checklist for Case Reports. Non-randomized comparative studies were assessed using the Risk Of Bias In Non-randomized Studies of Interventions (ROBINS-I) tool (Sterne et al., 2016). The comparative study by Ferretti 2002 was assessed using ROBINS-I because participants were allocated alternately rather than through true randomization (Ferretti and Ripamonti, 2002). Risk-of-bias assessments were used to inform interpretation and certainty of evidence, but studies were not excluded solely on the basis of methodological quality.

The certainty of evidence for the four primary device-related outcomes—device exposure, infection, mechanical failure, and unplanned reoperation or device removal—was assessed using the Grading of Recommendations Assessment, Development and Evaluation (GRADE) approach (Guyatt et al., 2008). Because the evidence was derived predominantly from single-arm observational case series, the initial certainty was rated as low. Potential downgrading considered study limitations, inconsistency, indirectness, imprecision, and publication bias, including concerns related to small studies and selective outcome reporting. Mechanical failure was considered particularly vulnerable to imprecision because few events were observed. Graft consolidation or continuity restoration was not assessed as a formal primary GRADE outcome because its definitions and reporting varied across studies.

Overall certainty was categorized as high, moderate, low, or very low. Interpretation emphasized the non-comparative nature of the pooled proportions, the predominance of small retrospective case series, sparse events for some outcomes, and the limited ability to estimate between-study heterogeneity reliably.

### 2.8 Statistical analysis

The primary synthesis was conducted as a single-arm proportional meta-analysis of device exposure, infection, mechanical failure, and unplanned reoperation or device removal. Analyses were performed in R version 4.6.0 using the metafor package. The primary model was a logistic-normal random-effects generalized linear mixed model fitted using rma. glmm with a logit-transformed proportion measure (Stijnen et al., 2010). This approach models the binomial likelihood directly and allows zero-event studies to be retained without applying continuity corrections.

For each outcome, pooled proportions and 95% confidence intervals were calculated. Between-study variance was estimated using τ^2^, and statistical heterogeneity was summarized using I^2^. Because few cohorts contributed to each analysis and several outcomes contained sparse events, I^2^ values of 0% and τ^2^ estimates of 0 were interpreted as indicating that between-study heterogeneity could not be estimated reliably rather than as evidence of true homogeneity. Prediction intervals were considered only when between-study variance could be meaningfully estimated and were not emphasized when τ^2^ was estimated as 0.

Sensitivity analyses included Freeman–Tukey double-arcsine-transformed random-effects models, fixed-effect models for reference, and leave-one-out analyses when at least five cohorts contributed to an outcome. A sensitivity analysis additionally included Onodera 2023, which met the other eligibility criteria but lacked sufficient information to confirm the minimum six-month follow-up criterion. An additional sensitivity analysis excluded Miyamoto 2014, the only cohort contributing reconstruction-site-level denominators, to evaluate the influence of combining patient-level and reconstruction-site-level units. Patient-level conversion was not performed because outcome-specific patient-level event counts could not be established unambiguously. If the primary generalized linear mixed model failed to converge or produced an unstable estimate, the fallback approach was Freeman–Tukey random-effects synthesis or descriptive synthesis, depending on data sparsity and interpretability.

Exploratory analyses addressed graft consolidation or mandibular continuity restoration, dental implant placement, prosthetic rehabilitation, and manufacturing approach. Exploratory proportional synthesis was considered for outcomes reported by at least three independent cohorts with extractable event counts and denominators; outcomes reported by fewer cohorts or with definitions considered too heterogeneous for pooling were summarized narratively. Manufacturing approach was categorized as additively manufactured patient-specific titanium mesh/tray systems *versus* non-additively manufactured systems, including preformed, stock, manually adapted, model-bent, cast, and other customized constructs produced without metal additive manufacturing. Because only two cohorts used additively manufactured systems and their outcome reporting differed, manufacturing comparisons were descriptive and exploratory; formal between-group inference was not performed.

Publication-bias tests and funnel-plot asymmetry assessments were not performed because fewer than 10 cohorts contributed to each pooled outcome. All pooled estimates were interpreted as non-comparative proportions rather than comparative treatment effects.

## 3 Results

### 3.1 Study selection

The literature searches identified 1,529 records from PubMed, Embase, Web of Science, Scopus, the Cochrane Central Register of Controlled Trials, and ClinicalTrials.gov. After removal of 793 duplicates, 736 unique records underwent title-and-abstract screening. All 11 records identified through ClinicalTrials.gov were duplicates of records retrieved from the bibliographic databases and did not contribute any independently identified record to screening. Inter-reviewer agreement at this stage was substantial (Cohen’s κ = 0.83).

**FIGURE 1:**
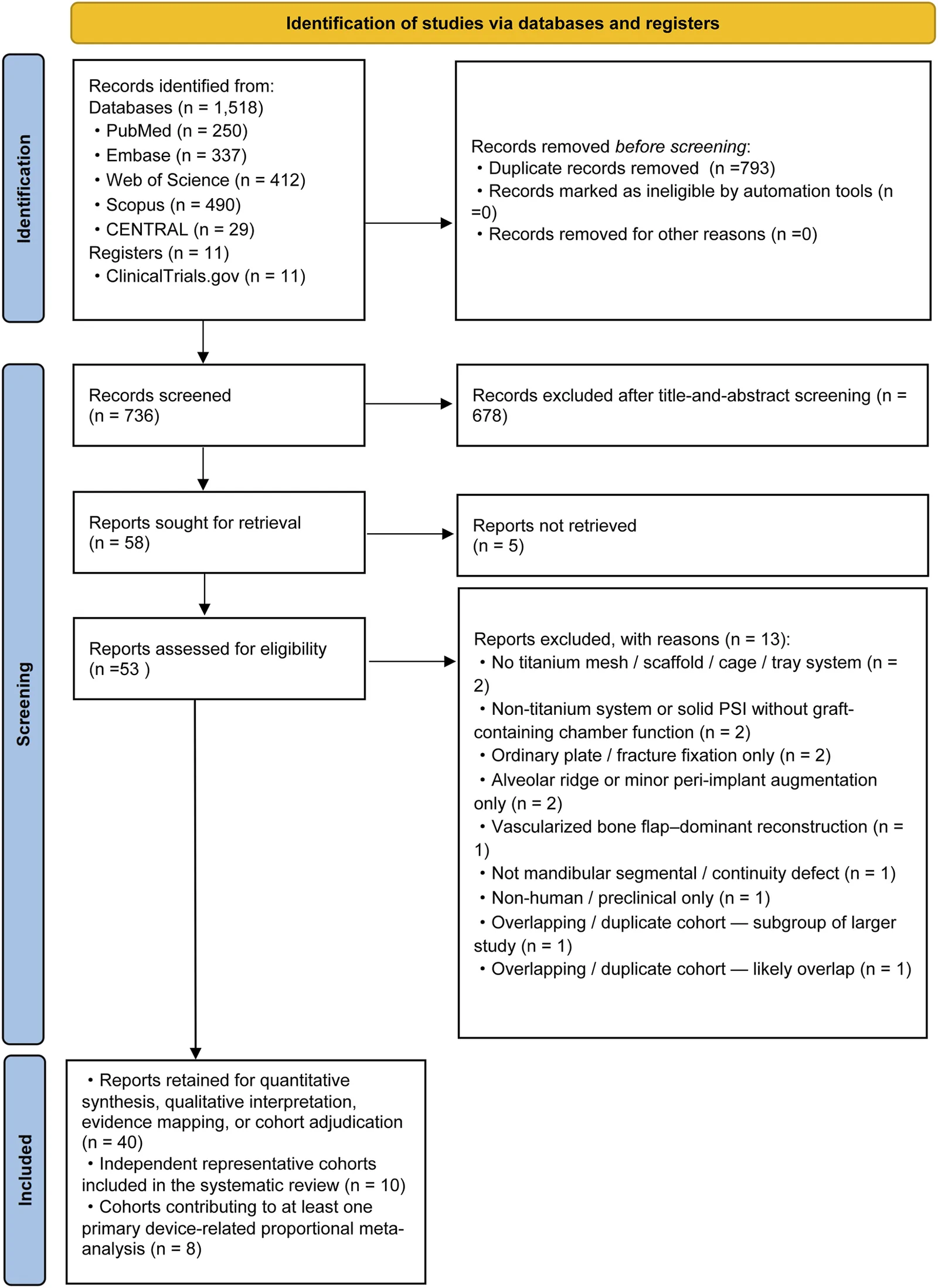
PRISMA 2020 flow diagram of study selection. All 11 records identified through ClinicalTrials.gov were duplicates of records retrieved from the bibliographic databases and did not contribute any independently identified records to screening. Abbreviations: CENTRAL, Cochrane Central Register of Controlled Trials; PSI, patient-specific implant.

After title-and-abstract screening, 678 records were excluded and 58 reports were sought for full-text retrieval. Five reports could not be retrieved, leaving 53 reports for full-text eligibility assessment. Thirteen reports were excluded with reasons, as shown in Figure 1. The remaining 40 reports were retained for quantitative synthesis, qualitative interpretation, evidence mapping, or cohort adjudication, as appropriate.

Following full-text assessment and cohort adjudication, 10 representative independent cohorts were included in the systematic review. Eight cohorts contributed data to at least one primary device-related proportional meta-analysis. Two additional cohorts did not contribute to the primary pooled estimates: Ferretti 2002 was retained for contextual and exploratory interpretation, whereas Onodera 2023 was included only in sensitivity analyses because the reported follow-up information was insufficient to confirm the minimum six-month criterion. Depending on outcome availability, denominator separability, and primary-analysis eligibility, five to seven cohorts contributed to each primary pooled analysis. Overlapping, historical, technical, or non-separable reports were retained only for supplementary interpretation, evidence mapping, or cohort adjudication and were not counted again in the quantitative synthesis. The study-selection process is shown in Figure 1.

### 3.2 Study characteristics

The core characteristics of the 10 representative cohorts are summarized in Table 1, with detailed clinical and methodological characteristics provided in Supplementary Table S2. The evidence base consisted predominantly of small clinical cohorts published from the early 2000s to the 2020s. Several cohorts originated from Japanese institutions, and the overall evidence base was geographically and institutionally concentrated.

**TABLE 1:** Core characteristics of the 10 representative independent cohorts included in the systematic review.

Author, year	Country (centre)	Study design	Reported cohort size	Eligible denominator	Device/manufacturing approach	Graft material	Follow-up
Yamada 2016	Japan (tsurumi University)	Retrospective case series	21	19	Custom titanium mesh tray; non-AM	PCBM	Mean 29 months
Onodera 2023	Japan (iwate medical University)	Retrospective case series	18	12	Custom titanium mesh; non-AM	PCBM	Short-term; exact follow-up duration not extractable
Danjo 2025	Japan (saga University)	Retrospective case series (long-term)	21	21	Preformed titanium mesh tray (Dumbach/OsteoMed); non-AM	PCBM ± block graft	Post-prosthetic observation: Mean 150.7 months
Miyamoto 2014	Japan (kyushu dental University)	Retrospective case series	9 patients	10 reconstruction sites	Titanium mesh tray; non-AM	PCBM	Mean 83.2 months
Iino 2009 (dumbach)	Japan (multicentre: Tokyo/Akita/Tsurumi)	Multi-institution retrospective case series	15	15	Dumbach titanium mesh-tray; non-AM	PCBM; PRP in some	Mean 62.7 months
Shehab 2018	Egypt (cairo University)	Prospective case series	8	8	Patient-specific titanium tray; AM (EBM)	Particulate bone + marrow (PCBM-equivalent); PRF	1 year
Sato 2022	Japan (showa University)	Retrospective case series	5	5	Custom titanium mesh cage; non-AM	PCBM	Mean 44.6 months
Zhou 2010	China (fourth military medical Univ., Xi’an)	Prospective case series	6	6	Customized cast titanium tray; non-AM	Particulate cancellous bone + marrow (PCBM-equivalent); cortical blocks	Mean 50 months
Zhao 2023	China (shanghai ninth People’s hospital)	Prospective case series	7	7	Patient-specific titanium mesh; AM (SLM)	Cancellous bone; marrow not specified	6 months
Ferretti 2002	South Africa (University of the Witwatersrand)	Prospective alternate-allocation comparative study	13	13	Preformed titanium mesh tray; non-AM	Corticocancellous block ± bovine BMP/DBM; no PCBM	13 months–4 years

AM, was defined as additively manufactured patient-specific metal systems; only Shehab 2018 and Zhao 2023 were classified as AM., Eligible denominator refers to the segmental or continuity-defect subgroup after cohort adjudication. Miyamoto 2014 included nine patients and 10 reconstruction sites; primary device-related outcomes were reported using reconstruction-site-level denominators. Onodera 2023 was retained in the systematic review but excluded from the primary pooled estimates because the reported follow-up information was insufficient to confirm the minimum six-month criterion.

For Danjo 2025, the reported mean of 150.7 months (range 4 months–27, years 11 months) refers to observation after delivery of the implant-supported prosthesis; reconstruction-level follow-up exceeded six months in all patients. Abbreviations: AM, additively manufactured; BMP, bone morphogenetic protein; DBM, demineralized bone matrix; NR, not reported; PCBM, particulate cancellous bone and marrow; PRF, platelet-rich fibrin; PRP, platelet-rich plasma.

Nine cohorts were single-arm retrospective or cohort-like clinical series. Ferretti 2002 was a prospective comparative study using alternate allocation and was assessed separately using ROBINS-I. Clinical indications included benign and malignant tumours, trauma, infection-related bone loss, osteoradionecrosis, and failed previous reconstruction. Cohorts also differed in defect location and extent, immediate or delayed reconstruction, radiotherapy exposure, and reported soft-tissue reconstruction or coverage. Reporting of these clinical variables was incomplete in some studies; available cohort-level information and unreported fields are presented systematically in Supplementary Table S2.

Titanium mesh/tray systems were used as graft-containing reconstruction chambers to maintain the reconstructed space, contain bone graft material, and support mandibular contour. Particulate cancellous bone and marrow was used in the Japanese cohorts. Particulate bone-and-marrow grafting was also reported in Shehab 2018 and Zhou 2010, whereas Zhao 2023 used iliac cancellous bone with marrow status not specified. Other approaches included corticocancellous block grafting, bone substitutes, and osteogenic or biologic adjuncts. Graft types and adjunctive procedures are detailed in Supplementary Table S2.

Device design and manufacturing approach varied across cohorts. Non-additively manufactured systems included preformed, stock, manually adapted, model-bent, cast, and other customized titanium mesh/tray constructs. Only Shehab 2018 and Zhao 2023 used additively manufactured patient-specific titanium mesh/tray systems. Because the additively manufactured subgroup comprised only two cohorts with uneven outcome reporting, manufacturing comparisons were treated as descriptive and exploratory, and no formal between-group inference was performed.

Eligible denominators were defined according to the segmental or continuity mandibular reconstructions evaluated in each cohort. Total cohort size and eligible denominator differed in mixed cohorts when marginal defects, non-continuity reconstructions, or non-separable subgroups were present. Miyamoto 2014 included nine patients and 10 reconstruction sites; device-related outcomes from this cohort were reported using reconstruction-site-level denominators, whereas most other cohorts used patient-level denominators. The influence of this mixed denominator unit was examined in an additional sensitivity analysis reported in Supplementary Table S3. Cohort-specific denominator decisions and analytic contributions are summarized in Table 2.

**TABLE 2:** Cohort adjudication and denominator decisions.

Cohort/report	Cohort cluster/institution	Total n	Eligible denominator	Denominator unit	Representative-cohort decision	Primary pooled-analysis status	Main reason/adjudication note	Outcome-specific denominator notes	Related reports/overlap notes
Yamada 2016 (tsurumi)	Tsurumi University (tsurumi cluster)	21	19	Patient	Representative of the tsurumi cluster	Included in primary pooled estimates (where extractable)	Most complete tsurumi report; two marginal cases were excluded from the eligible segmental denominator	Eligible (segmental) denominator n = 19; outcome-specific contributions shown in Figure 2	Subsumes earlier or subset tsurumi reports; supplementary only and not double-counted
Onodera 2023 (iwate)	Iwate medical University	18	12	Patient	Representative iwate cohort; retained in the systematic review	Sensitivity analysis only; excluded from the primary pooled estimates	The reported follow-up information was insufficient to confirm the minimum 6-month criterion; six marginal-defect cases were excluded from the eligible denominator	Outcome-specific contributions are shown in Figure 2	Oyamada 2025 is a single case within this cohort; not double-counted
Danjo 2025 (saga)	Saga University	21	21	Patient	Representative saga cohort	Included in primary pooled estimates (where extractable)	All segmental; long-term cohort. Preformed titanium mesh tray (Dumbach/OsteoMed); PCBM ± block graft; non-AM.	Exposure was coded as COMB because it was reported only as part of a combined exposure/infection category and was not pooled as a separate exposure outcome; recurrence-related removal was excluded from device-related reoperation/removal	Subsumes earlier yamashita single-case reports; not double-counted
Miyamoto 2014 (kyushu)	Kyushu dental University	9 patients (10 sites)	10 reconstruction sites	Site (primary device-complication outcomes)	Independent kyushu cohort	Included in primary pooled estimates (where extractable)	Independent institution and device era	Primary device-related denominators were reconstruction-site level (10 sites); implant and prosthetic outcomes were reported per patient (n = 9). The influence of the reconstruction-site-level denominator was evaluated in Supplementary Table S3	No additional overlapping representative report identified
Iino 2009 (dumbach)	University of Tokyo/Akita/Tsurumi (multi-institution)	15	15	Patient	Independent earlier multi-institution dumbach cohort	Included in primary pooled estimates (where extractable)	Counted once as an independent earlier dumbach cohort because its recruitment period and ready-made device era were distinct from those of the later tsurumi cohort	Unplanned reoperation or device removal was non-separable and classified as NSE in Figure 2	Although the cohort included tsurumi, its 1999–2004 recruitment period and ready-made dumbach device era were distinct from the later tsurumi cohort represented by yamada 2016
Ferretti 2002	University of the Witwatersrand	13	13	Patient	Prospective alternate-allocation comparative study	Retained for contextual/exploratory interpretation; not included in primary pooled device-related estimates	Assessed with ROBINS-I because allocation was alternate rather than truly randomized	No device-related complication events or denominators contributed to the primary pools	Two arms: autogenous bone graft (n = 7) *versus* osteogenic device (n = 6); the same preformed titanium mesh tray was used in both arms
Shehab 2018	Cairo University	8	8	Patient	Independent cohort	Included in primary pooled estimates (where extractable)	AM patient-specific titanium mesh tray (electron beam melting)	Outcome-specific contributions shown in Figure 2	-
Zhou 2010	Fourth military medical University, Xi’an	6	6	Patient	Independent cohort	Included in primary pooled estimates (where extractable)	Customized cast titanium tray (RE/CAD/RP resin model followed by investment casting); non-AM.	Outcome-specific contributions shown in Figure 2	-
Zhao 2023	Shanghai Ninth People’s hospital	7	7	Patient	Independent cohort	Included in primary pooled estimates (where extractable)	AM patient-specific titanium mesh (selective laser melting)	Coarse complication reporting; outcome-specific contributions shown in Figure 2	-
Sato 2022 (showa)	Showa University	5	5	Patient	Independent cohort	Included in primary pooled estimates (where extractable)	Non-AM custom/model-bent titanium mesh cage	Reoperation/removal counted once; device removal was revision in the same patient. Outcome-specific contributions are shown in Figure 2	-

COMB, combined exposure/infection category; NSE, non-separable eligible denominator or events; ROBINS-I, Risk Of Bias In Non-randomized Studies of Interventions.

**FIGURE 2:**
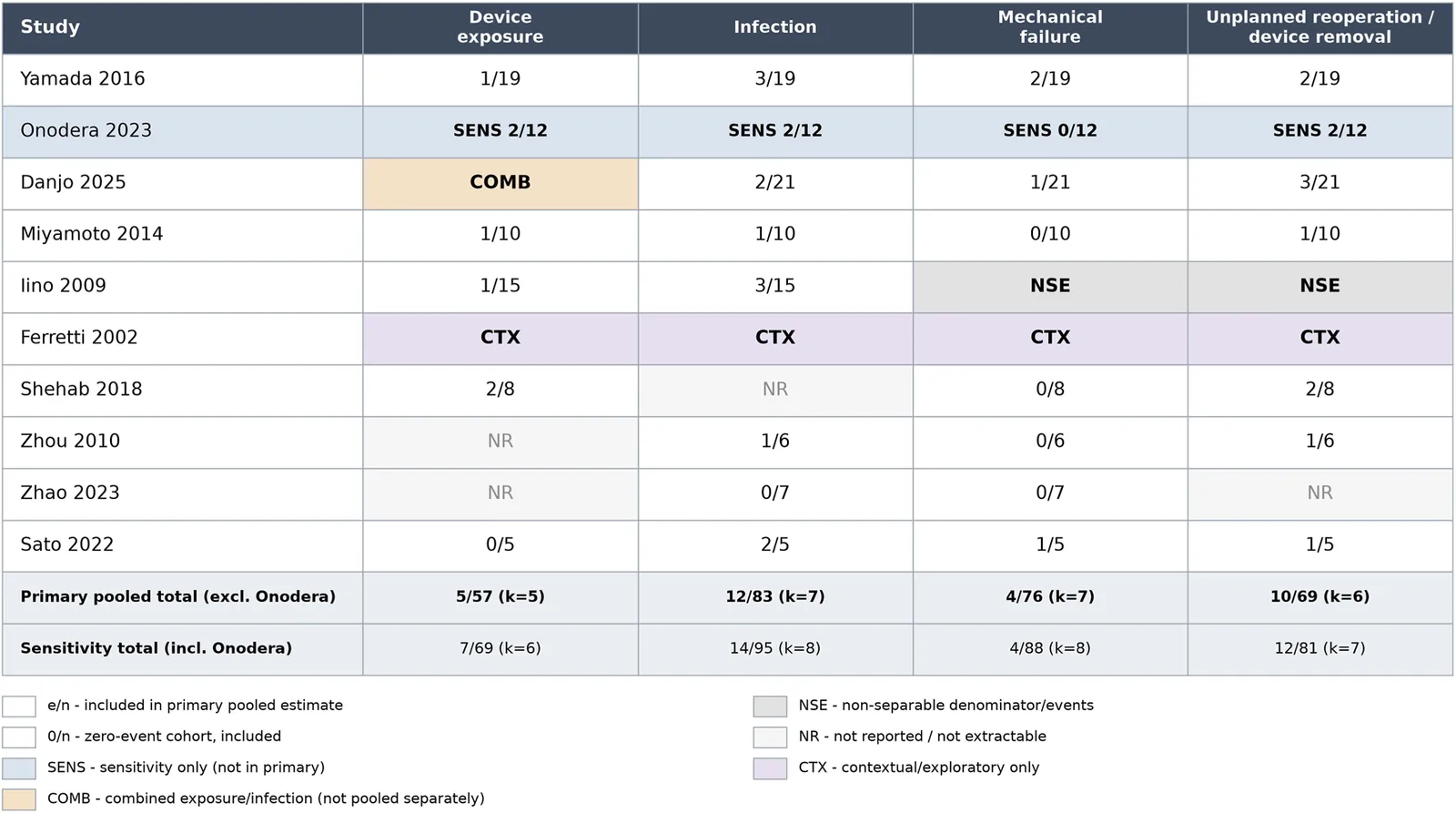
Study-by-outcome contribution matrix for the primary device-related outcomes.

This table summarizes representative-cohort selection, eligible denominators, denominator units, and primary pooled-analysis status. Outcome-specific cohort contributions are shown in Figure 2.

### 3.3 Cohort adjudication and outcome-specific contribution

Cohort-level adjudication was required because several reports shared institutions, author groups, device platforms, or partially overlapping clinical contexts. Representative independent cohorts were selected to avoid double-counting, while related reports were retained for qualitative interpretation, evidence mapping, or clarification of cohort overlap when relevant. Cohort-adjudication and denominator decisions are summarized in Table 2, and study-level contributions to the primary outcomes are shown in Figure 2.

Yamada 2016 was selected as the representative Tsurumi cohort, with 19 eligible patients after exclusion of two marginal-defect cases. Onodera 2023 represented the Iwate cohort, with 12 eligible segmental cases among 18 patients; however, the reported follow-up information was insufficient to confirm the minimum six-month criterion. This cohort was therefore excluded from the primary pooled estimates and included only in sensitivity analyses. Danjo 2025 represented the Saga cohort, with all 21 patients eligible. Device exposure was not entered as a separate outcome for this cohort because it was reported only as part of a combined exposure/infection category. Tumour recurrence-related device removal was excluded from the outcome of unplanned reoperation or device removal, whereas infection and mechanical failure were extractable for the corresponding primary analyses.

Miyamoto 2014 included nine patients and 10 reconstruction sites and contributed reconstruction-site-level denominators for the primary device-related outcomes. Iino 2009 was treated as an independent earlier multi-institution Dumbach cohort because its recruitment period and ready-made device era were distinct from those of the later Tsurumi cohort represented by Yamada 2016. Device exposure and infection were extractable from Iino 2009, whereas mechanical failure and unplanned reoperation or device removal were not separable according to the primary outcome definitions. Ferretti 2002 was retained for contextual and exploratory interpretation but did not contribute to the primary pooled device-related outcomes.

The Tokyo Medical reports by Matsuo and Iwamoto were reviewed but were not represented among the quantitative cohorts because no confirmed cases simultaneously met the eligibility criteria for a segmental or continuity mandibular defect and titanium mesh/tray reconstruction. These reports were retained only for contextual interpretation.

The remaining independent cohorts—Shehab 2018, Zhou 2010, Zhao 2023, and Sato 2022—contributed to individual outcome pools according to event-count and denominator extractability, with zero-event cohorts retained when eligible denominators were available. In the primary analyses excluding Onodera 2023, device exposure was informed by five cohorts with 5 events among 57 denominators, infection by seven cohorts with 12 events among 83 denominators, mechanical failure by seven cohorts with 4 events among 76 denominators, and unplanned reoperation or device removal by six cohorts with 10 events among 69 denominators. Denominator units were patients or reconstruction sites according to the original cohort-level reporting. The complete contribution pattern, including not-reported and non-separable outcomes, is shown in Figure 2.

### 3.4 Primary device-related outcomes

**FIGURE 3:**
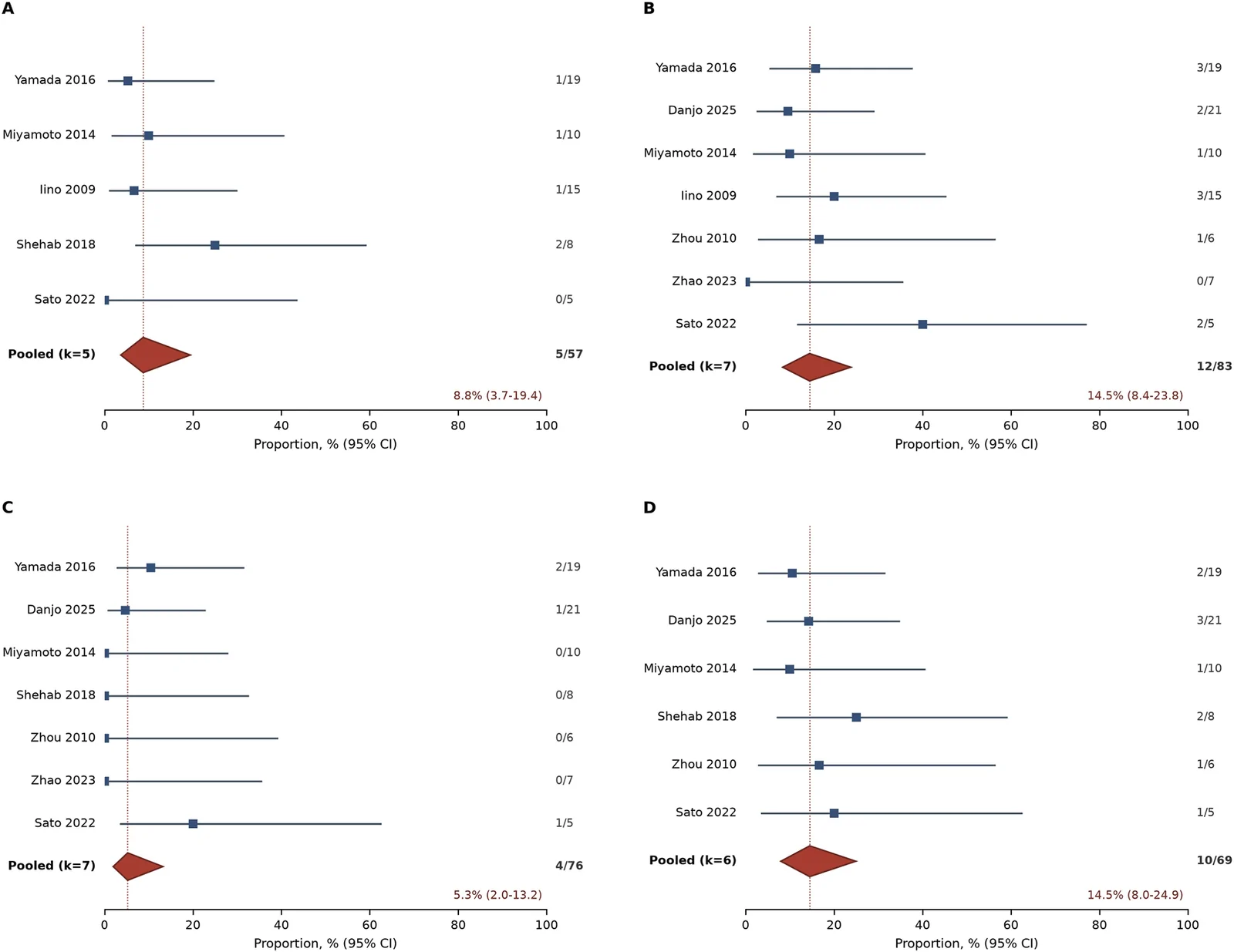
Forest plots of the primary device-related outcomes. Forest plots show pooled proportions for **(A)** device exposure **(B)** infection **(C)** mechanical failure, and **(D)** unplanned reoperation or device removal, estimated using the primary logistic-normal random-effects generalized linear mixed model. Squares represent cohort-level proportions, horizontal lines represent 95% confidence intervals, and diamonds represent pooled estimates. The primary analyses excluded Onodera 2023 because the reported follow-up information was insufficient to confirm the minimum six-month criterion. Sensitivity analyses including Onodera 2023 and excluding Miyamoto 2014 are presented in Supplementary Table S3.

The pooled estimates for the four primary device-related outcomes are shown in Figure 3 and Table 3. In the primary analyses excluding Onodera 2023, the pooled proportion of device exposure was 8.8% (95% CI, 3.7%–19.4%), infection was 14.5% (95% CI, 8.4%–23.8%), mechanical failure was 5.3% (95% CI, 2.0%–13.2%), and unplanned reoperation or device removal was 14.5% (95% CI, 8.0%–24.9%).

**TABLE 3:** Primary pooled estimates and certainty of evidence for the four device-related outcomes.

Outcome	Cohorts, k	Events/denominator	Pooled proportion, %	95% CI, %	I^2^, %	τ^2^	Certainty of evidence	Main reasons for certainty rating
Device exposure	5	5/57	8.8	3.7–19.4	0	0	Very low	Single-arm observational evidence; serious risk of bias and serious imprecision due to few cohorts and wide confidence intervals
Infection	7	12/83	14.5	8.4–23.8	0	0	Very low	Single-arm observational evidence; serious risk of bias and serious imprecision due to few cohorts and wide confidence intervals
Mechanical failure	7	4/76	5.3	2.0–13.2	0	0	Very low	Single-arm observational evidence; serious risk of bias and very serious imprecision due to sparse events and a fragile estimate
Unplanned reoperation or device removal	6	10/69	14.5	8.0–24.9	0	0	Very low	Single-arm observational evidence; serious risk of bias and serious imprecision due to few cohorts and wide confidence intervals

Primary analyses excluded Onodera 2023 because the reported follow-up information was insufficient to confirm the minimum six-month criterion. Pooled proportions were estimated using a logistic-normal random-effects generalized linear mixed model. I^2^ = 0% and τ^2^ = 0 were interpreted cautiously because the small number of cohorts and sparse events limited reliable estimation of between-study heterogeneity. Certainty of evidence was rated using GRADE., sensitivity analyses, including analyses incorporating Onodera 2023 and excluding the reconstruction-site-level cohort Miyamoto 2014, are provided in Supplementary Table S3.

Abbreviations: CI, confidence interval; GRADE, grading of recommendations assessment, Development and Evaluation; GLMM, generalized linear mixed model.

For all four outcomes, I^2^ was 0% and τ^2^ was estimated as 0. These values were interpreted cautiously because the small numbers of contributing cohorts and sparse events limited reliable estimation of between-study heterogeneity; they should not be interpreted as evidence of true homogeneity. Mechanical failure was the sparsest primary outcome and remained a fragile estimate despite its lower point estimate.

The certainty of evidence was rated as very low for all four primary outcomes. This reflected the predominantly small, non-randomized, single-arm evidence base, sparse events for some outcomes, imprecision, and limitations in outcome and denominator reporting.

### 3.5 Sensitivity and exploratory analyses

Sensitivity analyses are summarized in Supplementary Table S3. Including Onodera 2023 produced only small numerical changes: the pooled proportions were 10.1% (95% CI, 4.9%–19.8%) for device exposure, 14.7% (95% CI, 8.9%–23.4%) for infection, 4.5% (95% CI, 1.7%–11.5%) for mechanical failure, and 14.8% (95% CI, 8.6%–24.3%) for unplanned reoperation or device removal. Freeman–Tukey, fixed-effect, and leave-one-out analyses were directionally consistent with the primary GLMM estimates. Mechanical failure remained the least stable outcome because of the small number of events.

An additional sensitivity analysis excluded Miyamoto 2014, the cohort that contributed reconstruction-site-level denominators. The resulting pooled proportions were 8.5% (95% CI, 3.2%–20.6%) for device exposure, 15.1% (95% CI, 8.6%–25.2%) for infection, 6.1% (95% CI, 2.3%–15.1%) for mechanical failure, and 15.3% (95% CI, 8.1%–26.8%) for unplanned reoperation or device removal. Exclusion produced modest changes in point estimates and wider confidence intervals for all four outcomes, without changing their overall exploratory interpretation. I^2^ remained 0% and τ^2^ remained 0 in each analysis.

Manufacturing approach was explored using the strict grouping of additively manufactured patient-specific titanium mesh/tray systems *versus* non-additively manufactured systems. Only Shehab 2018 and Zhao 2023 used additively manufactured systems, and they contributed different combinations of outcomes. Formal subgroup pooling and between-group inference were therefore not considered appropriate. The crude event proportions for additively manufactured *versus* non-additively manufactured systems were 2 of 8 *versus* 3 of 49 for device exposure, 0 of 7 *versus* 12 of 76 for infection, 0 of 15 *versus* 4 of 61 for mechanical failure, and 2 of 8 *versus* 8 of 61 for unplanned reoperation or device removal. These descriptive comparisons should not be interpreted as evidence of differential performance by manufacturing approach.

Graft consolidation or mandibular continuity restoration was reported in four cohorts and yielded an exploratory pooled proportion of 80.8% (95% CI, 67.8%–89.3%). This estimate was considered hypothesis-generating because outcome definitions included heterogeneous radiographic, clinical, and composite success assessments. Dental implant placement was reported in seven cohorts, with an exploratory pooled proportion of 56.0% (95% CI, 23.7%–84.0%). This estimate was influenced by Danjo 2025, which was restricted to patients who reached implant-supported prosthetic loading; after excluding this cohort, the pooled proportion was 41.8% (95% CI, 22.9%–63.4%). Prosthetic rehabilitation was reported in seven cohorts, with an exploratory pooled proportion of 86.0% (95% CI, 66.6%–95.0%); after exclusion of Danjo 2025, the corresponding estimate was 78.6% (95% CI, 61.2%–89.6%). These downstream outcomes were interpreted cautiously because they depended on patient selection, staged treatment pathways, and reporting practices rather than device-related performance alone.

### 3.6 Risk of bias and certainty of evidence

Risk-of-bias assessments are summarized in Supplementary Table S4. The evidence base was dominated by small single-arm clinical series. The principal methodological concerns were retrospective or cohort-like designs, limited sample sizes, incomplete reporting of consecutive or complete inclusion, heterogeneous follow-up reporting, and non-standardized definitions of device-related outcomes. Several cohorts also required denominator adjudication because eligible segmental or continuity-defect reconstructions were embedded within broader clinical series or because outcome-specific events or denominators were not separately extractable. Ferretti 2002 was assessed using ROBINS-I because allocation was alternate rather than truly randomized; it was retained for contextual and exploratory interpretation but did not contribute to the primary pooled device-related outcomes.

The certainty of evidence for all four primary device-related outcomes was rated as very low (Table 3; Supplementary Table S5). This rating reflected the single-arm observational evidence base, risk-of-bias concerns inherent to small clinical series, sparse events, and imprecision due to few cohorts and wide confidence intervals. Mechanical failure was particularly fragile because only four events were available in the primary dataset. Although the sensitivity analyses produced only modest changes in the point estimates, they did not address the underlying limitations in study design, clinical heterogeneity, outcome reporting, denominator units, or event sparsity; the certainty ratings therefore remained very low.

## 4 Discussion

This systematic review and proportional meta-analysis provides a focused synthesis of device-related complications associated with titanium mesh/tray systems used with bone grafting for mandibular continuity reconstruction. Exposure, infection, and unplanned reoperation or device removal were the principal reported concerns, whereas mechanical failure was less frequently reported but was based on sparse events. The findings should be interpreted as exploratory clinical benchmarks rather than definitive estimates of comparative performance. They nevertheless emphasize the importance of case selection, stable graft containment, adequate soft-tissue coverage, and structured postoperative follow-up.

Titanium mesh/tray systems may be conceptualized as graft-containing reconstruction chambers rather than simple fixation hardware (Ikawa et al., 2016; Ripamonti and Petit, 2009; Iino et al., 2009; Hodgdon et al., 2018; Wang et al., 2021; Ferretti and Ripamonti, 2002). Their principal functions are to maintain a three-dimensional grafted space, contain particulate or block graft material, support mandibular contour, and provide mechanical stabilization through load sharing (Zhao et al., 2023; Onodera et al., 2023; Huang et al., 2023; Ostrovidov et al., 2023; Wang et al., 2021; Ferretti and Ripamonti, 2002). Continuity restoration depends primarily on the graft material, recipient bed, soft-tissue envelope, and local healing conditions, whereas the titanium construct provides structural containment and support (Iino et al., 2009; Ostrovidov et al., 2023; Ferretti and Ripamonti, 2002). This distinction is important because the titanium device should not be interpreted as intrinsically osteogenic or osteoinductive.

**FIGURE 4:**
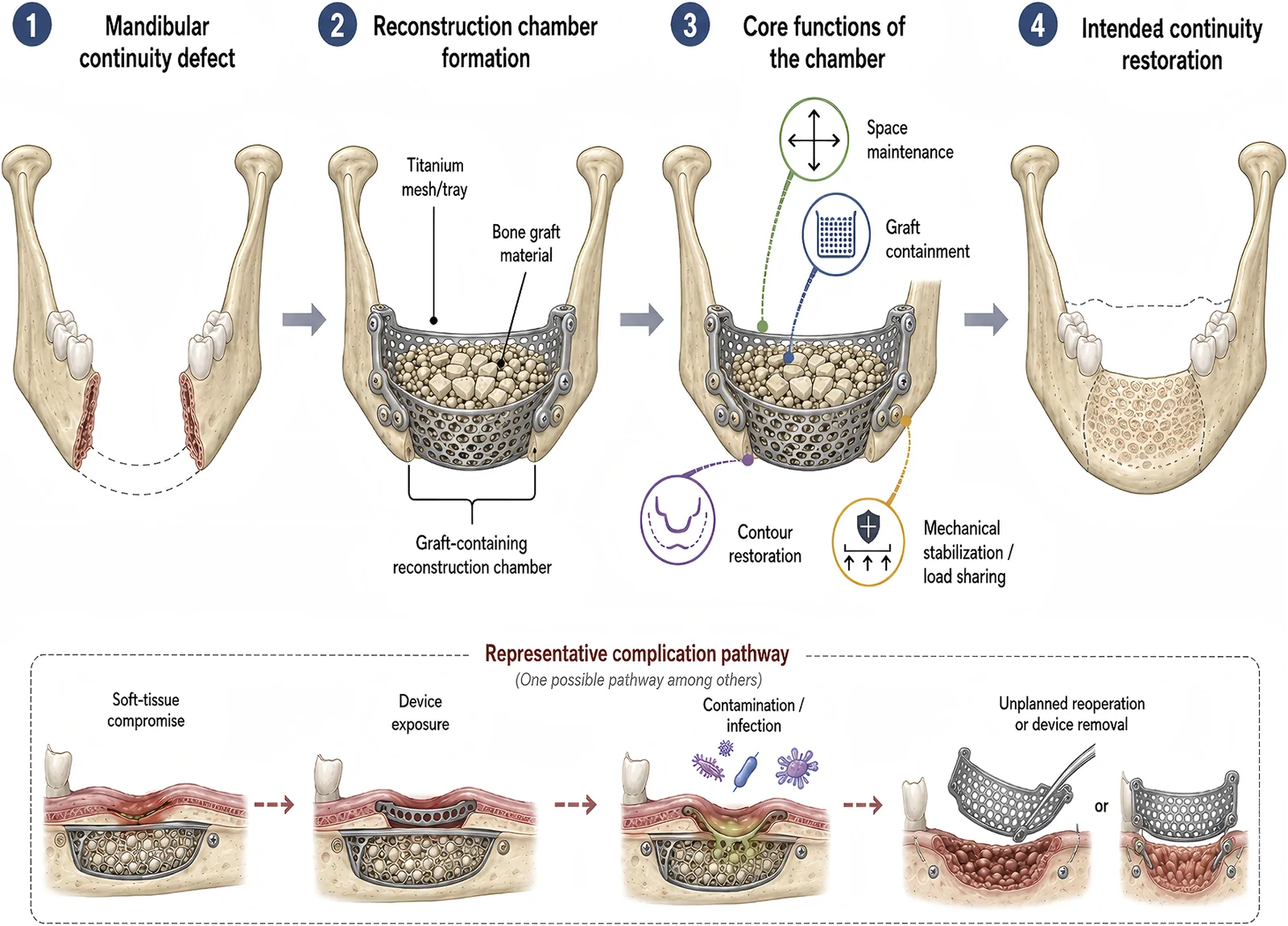
Conceptual mechanism of the titanium mesh/tray graft-containing reconstruction chamber and a representative device-related complication pathway.

The chamber-based concept also provides a framework for interpreting the observed complication pattern. Compromise of the soft-tissue envelope may lead to device exposure and loss of separation between the graft-containing chamber and the oral or external environment, potentially increasing susceptibility to contamination, infection, and subsequent reoperation or device remova (Hodgdon et al., 2018; Zhao et al., 2023; Ostrovidov et al., 2023; Ferretti and Ripamonti, 2002). These events do not necessarily occur as a fixed causal sequence, and the primary outcomes were analysed independently. Figure 4 therefore illustrates a representative complication pathway rather than an inevitable progression from exposure to infection or removal.

Manufacturing approach remains insufficiently evaluated in the clinical literature (Hodgdon et al., 2018; Hoefert et al., 2026; Zhao et al., 2023; Onodera et al., 2023; Huang et al., 2023). Additively manufactured patient-specific titanium mesh/tray systems may theoretically improve anatomical fit, contour planning, and correspondence between the planned reconstruction and the implanted construct, whereas preformed, stock, model-bent, cast, and manually adapted systems depend more heavily on model-based or intraoperative adaptation (Ikawa et al., 2016; Iino et al., 2009; Hodgdon et al., 2018; Onodera et al., 2023; Ostrovidov et al., 2023). However, only two cohorts in this review used additively manufactured systems, and their outcome reporting was sparse and uneven (Zhao et al., 2023). The available descriptive comparisons therefore cannot establish whether additive manufacturing improves or worsens device-related outcomes. Future evaluations should distinguish manufacturing method from other potentially influential factors, including defect complexity, fixation strategy, chamber geometry, graft type, soft-tissue coverage, and institutional experience.

Titanium mesh/tray systems should be positioned within the broader range of mandibular reconstructive options, which differ in defect complexity, vascularity, soft-tissue requirements, donor-site considerations, and rehabilitation goals (Ruf et al., 2025; Brown et al., 2017). They should not be regarded as direct substitutes for vascularized bone flaps in extensive composite defects, heavily irradiated fields, poor soft-tissue conditions, or situations requiring immediate vascularized bone and soft-tissue replacement (Ruf et al., 2025; Brown et al., 2017; Ikawa et al., 2016). Rather, they may be considered in selected clinical contexts in which the defect, recipient bed, available soft-tissue coverage, graft supply, and staged rehabilitation plan are favourable (Ikawa et al., 2016; Ripamonti and Petit, 2009; Iino et al., 2009; Zhao et al., 2023; Ferretti and Ripamonti, 2002). Because the present evidence is non-comparative, the pooled proportions cannot determine whether this approach performs better or worse than flap-based reconstruction, solid patient-specific implants, or other grafting strategies.

The observed complication pattern highlights practical considerations for reconstructive planning and surveillance. Stable fixation and graft containment, chamber geometry that minimizes soft-tissue tension, and early recognition of mucosal breakdown, exposure, drainage, or infection may be particularly relevant (Iino et al., 2009; Huang et al., 2023; Wang et al., 2021; Ferretti and Ripamonti, 2002). Dental implant placement and prosthetic rehabilitation also indicate that these procedures form part of a staged reconstructive pathway rather than an isolated bony intervention (Onodera et al., 2023; Huang et al., 2023; Ostrovidov et al., 2023). These downstream outcomes depend on patient selection, graft consolidation, implant feasibility, prosthetic planning, and the ability to complete multiple stages of treatment; they should not be interpreted as direct measures of device performance.

Several limitations should be considered. First, the evidence was derived predominantly from single-arm, non-comparative clinical series, precluding causal inference and reliable comparison with alternative reconstructive approaches. Second, the cohorts were small and geographically or institutionally concentrated, with substantial representation from Japanese series using particular PCBM-based grafting protocols and device eras. The applicability of the pooled estimates to other healthcare systems, defect patterns, soft-tissue strategies, device designs, and grafting protocols is therefore uncertain. Third, certainty was very low for all primary device-related outcomes. Although the sensitivity analyses produced only modest numerical changes in the point estimates, they did not address the underlying limitations related to study design, clinical heterogeneity, event sparsity, outcome reporting, and denominator units. Future research should use prospective, multi-institutional designs with standardized definitions of device exposure, infection, mechanical failure, reoperation or device removal, graft consolidation, and rehabilitation outcomes. Detailed reporting of defect location and extent, reconstruction timing, radiotherapy, soft-tissue management, graft composition, denominator units, and manufacturing approach will be necessary to support more informative synthesis and appropriately selected comparative studies. Cells show event counts and denominators for cohorts contributing to each outcome or the applicable evidence-status code. Primary pooled analyses excluded Onodera 2023 because the reported follow-up information was insufficient to confirm the minimum six-month criterion; this cohort contributed only to sensitivity analyses. Miyamoto 2014 reported device-related outcomes using reconstruction-site-level denominators, and an additional sensitivity analysis excluding this cohort is presented in Supplementary Table S3. Abbreviations: COMB, combined exposure/infection category not pooled as a separate exposure outcome; CTX, contextual or exploratory evidence not included in the primary pooled estimates; NR, not reported or not extractable; NSE, non-separable eligible denominator or events; SENS, contribution to sensitivity analyses only.

## 5 Conclusion

This systematic review and proportional meta-analysis provides exploratory quantitative benchmarks for device-related complications associated with titanium mesh/tray systems used with bone grafting for mandibular continuity reconstruction. These systems may be considered in selected clinical contexts, but exposure, infection, and unplanned reoperation or device removal remain important concerns. The available evidence is derived mainly from small, single-arm, institutionally concentrated cohorts and is of very low certainty. Prospective multi-institutional studies with standardized clinical and outcome reporting are needed to define more clearly the role of titanium mesh/tray systems within mandibular reconstruction. The upper panels illustrate the conceptual sequence from a mandibular continuity defect to reconstruction chamber formation, the core structural functions of the chamber, and the intended restoration of mandibular continuity. The titanium mesh/tray system is intended to provide space maintenance, graft containment, contour restoration, and mechanical stabilization through load sharing rather than full load bearing. Panel 4 depicts the reconstructive goal; actual graft consolidation and continuity restoration varied across cohorts and were summarized as exploratory outcomes. The lower panels illustrate one possible complication pathway in which soft-tissue compromise may be followed by device exposure, contamination or infection, and unplanned reoperation or device removal. The primary outcomes were analysed as independent proportions and should not be interpreted as a fixed causal sequence. Particulate graft material is shown as a representative example; block grafts were also used in some cohorts.

## Data Availability

The original contributions presented in the study are included in the article/Supplementary Material, further inquiries can be directed to the corresponding author.

## References

[B1] Booth A. Clarke M. Dooley G. Ghersi D. Moher D. Petticrew M. (2012). The nuts and bolts of PROSPERO: an international prospective register of systematic reviews. Syst. Rev. 1, 2. 10.1186/2046-4053-1-2 22587842 PMC3348673

[B2] Brown J. S. Lowe D. Kanatas A. Schache A. (2017). Mandibular reconstruction with vascularised bone flaps: a systematic review over 25 years. Br. J. Oral Maxillofac. Surg. 55, 113–126. 10.1016/j.bjoms.2016.12.010 28065645

[B3] DeGould M. D. Goldberg J. S. (1991). Recurrence of an odontogenic keratocyst in a bone graft. Report of a case. Int. J. Oral Maxillofac. Surg. 20, 9–11. 10.1016/s0901-5027(05)80686-1 2019786

[B4] Farid Shehab M. Hamid N. M. A. Askar N. A. Elmardenly A. M. (2018). Immediate mandibular reconstruction via patient-specific titanium mesh tray using electron beam melting/CAD/rapid prototyping techniques: one-year follow-up. Int. J. Med. Robot. 14, e1895. 10.1002/rcs.1895 29464889

[B5] Ferretti C. Ripamonti U. (2002). Human segmental mandibular defects treated with naturally derived bone morphogenetic proteins. J. Craniofac Surg. 13, 434–444. 10.1097/00001665-200205000-00014 12040215

[B6] Guyatt G. H. Oxman A. D. Vist G. E. Kunz R. Falck-Ytter Y. Alonso-Coello P. (2008). GRADE: an emerging consensus on rating quality of evidence and strength of recommendations. Bmj 336, 924–926. 10.1136/bmj.39489.470347.AD 18436948 PMC2335261

[B7] Hodgdon T. Danrad R. Patel M. J. Smith S. E. Richardson M. L. Ballard D. H. (2018). Logistics of three-dimensional printing: primer for radiologists. Acad. Radiol. 25, 40–51. 10.1016/j.acra.2017.08.003 29030283 PMC6467477

[B8] Hoefert S. Emmler A. Naros A. Haefner J. Schoenhof R. (2026). Distress monitoring during surgical treatment of patients with primary head and neck tumors of oral cavity and oropharynx: a prospective pilot study. Int. J. Oral Maxillofac. Surg. 10.1016/j.ijom.2026.06.012 42321116

[B9] Huang S. Wei H. Li D. (2023). Additive manufacturing technologies in the oral implant clinic: a review of current applications and progress. Front. Bioeng. Biotechnol. 11, 1100155. 10.3389/fbioe.2023.1100155 36741746 PMC9895117

[B10] Iino M. Fukuda M. Nagai H. Hamada Y. Yamada H. Nakaoka K. (2009). Evaluation of 15 mandibular reconstructions with dumbach Titan mesh-system and particulate cancellous bone and marrow harvested from bilateral posterior ilia. Oral Surg. Oral Med. Oral Pathol. Oral Radiol. Endod. 107, e1–e8. 10.1016/j.tripleo.2008.12.018 19201629

[B11] Ikawa T. Shigeta Y. Hirabayashi R. Hirai S. Hirai K. Harada N. (2016). Computer assisted mandibular reconstruction using a custom-made Titan mesh tray and removable denture based on the top-down treatment technique. J. Prosthodont Res. 60, 321–331. 10.1016/j.jpor.2016.01.009 26895971

[B12] Lasserson T. J. Thomas J. Higgins J. P. (2019). Starting a review. Cochrane Handb. Syst. Rev. Interventions, 1–12. 10.1002/9781119536604.ch1

[B13] May S. M. (1994). Modelling observer agreement--an alternative to kappa. J. Clin. Epidemiol. 47, 1315–1324. 10.1016/0895-4356(94)90137-6 7722568

[B14] Miyamoto I. Yamashita Y. Yamamoto N. Nogami S. Yamauchi K. Yoshiga D. (2014). Evaluation of mandibular reconstruction with particulate cancellous bone marrow and titanium mesh after mandibular resection due to tumor surgery. Implant Dent. 23, 108–115. 10.1097/ID.0000000000000041 24637525

[B15] Munn Z. Barker T. H. Moola S. Tufanaru C. Stern C. McArthur A. (2020). Methodological quality of case series studies: an introduction to the JBI critical appraisal tool. JBI Evid. Synth. 18, 2127–2133. 10.11124/JBISRIR-D-19-00099 33038125

[B16] Munn Z. Stone J. C. Aromataris E. Klugar M. Sears K. Leonardi-Bee J. (2023). Assessing the risk of bias of quantitative analytical studies: introducing the vision for critical appraisal within JBI systematic reviews. JBI Evid. Synth. 21, 467–471. 10.11124/JBIES-22-00224 36476419

[B17] Onodera K. Miyamoto I. Hoshi I. Kawamata S. Takahashi N. Shimazaki N. (2023). Towards optimum mandibular reconstruction for dental occlusal rehabilitation: from preoperative virtual surgery to autogenous particulate cancellous bone and marrow graft with custom-made titanium Mesh-A retrospective study. J. Clin. Med. 12, 1122. 10.3390/jcm12031122 36769770 PMC9918119

[B18] Ostrovidov S. Ramalingam M. Bae H. Orive G. Fujie T. Shi X. (2023). Bioprinting and biomaterials for dental alveolar tissue regeneration. Front. Bioeng. Biotechnol. 11, 991821. 10.3389/fbioe.2023.991821 37122863 PMC10140526

[B19] Page M. J. McKenzie J. E. Bossuyt P. M. Boutron I. Hoffmann T. C. Mulrow C. D. (2021). The PRISMA 2020 statement: an updated guideline for reporting systematic reviews. Bmj 372, n71. 10.1136/bmj.n71 33782057 PMC8005924

[B20] Rethlefsen M. L. Kirtley S. Waffenschmidt S. Ayala A. P. Moher D. Page M. J. (2021). PRISMA-S: an extension to the PRISMA statement for reporting literature searches in systematic reviews. J. Med. Libr. Assoc. 109, 174–200. 10.5195/jmla.2021.962 34285662 PMC8270366

[B21] Ripamonti U. Petit J. C. (2009). Bone morphogenetic proteins, cementogenesis, myoblastic stem cells and the induction of periodontal tissue regeneration. Cytokine Growth Factor Rev. 20, 489–499. 10.1016/j.cytogfr.2009.10.016 19897401

[B22] Ruf P. Duda K. Fenske J. Orassi V. Lampert P. Kreiker H. (2025). Prospective functional assessment after mandible reconstruction and bone healing assessment. Clin. Oral Investig. 30, 14. 10.1007/s00784-025-06698-3 41410891 PMC12715053

[B23] Sterne J. A. Hernán M. A. Reeves B. C. Savović J. Berkman N. D. Viswanathan M. (2016). ROBINS-I: a tool for assessing risk of bias in non-randomised studies of interventions. Bmj 355, i4919. 10.1136/bmj.i4919 27733354 PMC5062054

[B24] Stijnen T. Hamza T. H. Ozdemir P. (2010). Random effects meta-analysis of event outcome in the framework of the generalized linear mixed model with applications in sparse data. Stat. Med. 29, 3046–3067. 10.1002/sim.4040 20827667

[B25] Thomas J. Kneale D. McKenzie J. E. Brennan S. E. Bhaumik S. (2019). Determining the scope of the review and the questions it will address. Cochrane Handb. Syst. Rev. Interventions, 13–31. 10.1002/9781119536604.ch2

[B26] Wang H. Yuan C. Lin K. Zhu R. Zhang S. (2021). Modifying a 3D-Printed Ti6Al4V implant with polydopamine coating to improve BMSCs growth, osteogenic differentiation, and *in situ* osseointegration *in vivo* . Front. Bioeng. Biotechnol. 9, 761911. 10.3389/fbioe.2021.761911 34926418 PMC8678591

[B27] Yamada H. Nakaoka K. Sonoyama T. Kumagai K. Ikawa T. Shigeta Y. (2016). Clinical usefulness of mandibular reconstruction using custom-made titanium mesh tray and autogenous particulate cancellous bone and marrow harvested from tibia and/or ilia. J. Craniofac Surg. 27, 586–592. 10.1097/SCS.0000000000002472 27092909

[B28] Zhao Z. Shen S. Li M. Shen G. Ding G. Yu H. (2023). Three-dimensional printed titanium mesh combined with iliac cancellous bone in the reconstruction of mandibular defects secondary to ameloblastoma resection. BMC Oral Health 23, 681. 10.1186/s12903-023-03386-0 37730602 PMC10510271

[B29] Zhou L. B. Shang H. T. He L. S. Bo B. Liu G. C. Liu Y. P. (2010). Accurate reconstruction of discontinuous mandible using a reverse engineering/computer-aided design/rapid prototyping technique: a preliminary clinical study. J. Oral Maxillofac. Surg. 68, 2115–2121. 10.1016/j.joms.2009.09.033 20542365

