# Supplementary material for: Titanium mesh and tray systems for mandibular continuity reconstruction with bone grafting: a systematic review and proportional meta-analysis of device-related complications
Source: Front Bioeng Biotechnol. 2026 Aug 26;14:1915266. doi: 10.3389/fbioe.2026.1915266 (PMC13553891; doi:10.3389/fbioe.2026.1915266)
Supplement: Supplementary file 1 [file DataSheet1.pdf]

Supplementary Table S1. Search strategies

Searches were conducted from database inception to 20 June 2026. No restrictions on publication year, language, or publication status were applied.

Database 1. PubMed

Records identified: 250

(("Mandible"[Mesh] OR mandib*[tiab] OR mandible[tiab] OR jaw[tiab] OR maxillofacial[tiab])
AND
(reconstruct*[tiab] OR defect*[tiab] OR continuity[tiab] OR segmental[tiab] OR mandibulectom*[tiab] OR hemimandibulectom*[tiab])
AND
("Titanium"[Mesh] OR titanium[tiab] OR TiMesh[tiab] OR "Ti mesh"[tiab])
AND
(mesh[tiab] OR meshes[tiab] OR scaffold*[tiab] OR cage*[tiab] OR tray[tiab] OR trays[tiab] OR grid[tiab] OR grids[tiab] OR "titanium mesh"[tiab] OR "titanium mesh tray"[tiab] OR "titanium scaffold"[tiab] OR "titanium scaffolds"[tiab] OR "titanium cage"[tiab] OR "titanium cages"[tiab] OR "titanium tray"[tiab] OR "titanium trays"[tiab] OR "mesh tray"[tiab] OR "mesh trays"[tiab] OR "titanium grid"[tiab] OR "porous titanium"[tiab] OR "lattice titanium"[tiab])
AND
("patient-specific"[tiab] OR "patient specific"[tiab] OR customized[tiab] OR customised[tiab] OR customize[tiab] OR customise[tiab] OR "custom-made"[tiab] OR "custom made"[tiab] OR personalized[tiab] OR personalised[tiab] OR "CAD/CAM"[tiab] OR "CAD-CAM"[tiab] OR "computer-aided design"[tiab] OR "computer aided design"[tiab] OR "computer-assisted"[tiab] OR "computer assisted"[tiab] OR "virtual surgical planning"[tiab] OR "3D printing"[tiab] OR "3D printed"[tiab] OR "3D-printed"[tiab] OR "three-dimensional printing"[tiab] OR "three-dimensional printed"[tiab] OR "additive manufacturing"[tiab] OR "additively manufactured"[tiab] OR "selective laser melting"[tiab] OR SLM[tiab] OR "electron beam melting"[tiab] OR EBM[tiab] OR "direct metal laser sintering"[tiab] OR DMLS[tiab] OR PCBM[tiab] OR "particulate cancellous bone"[tiab] OR "bone marrow"[tiab] OR "bone graft"[tiab] OR "bone grafts"[tiab] OR "bone grafting"[tiab] OR "bone regeneration"[tiab] OR "bone bridging"[tiab]))

Database 2. Embase (Embase.com)

Records identified: 337

('mandible'/exp OR mandib*:ti,ab,kw OR mandible:ti,ab,kw OR jaw:ti,ab,kw OR maxillofacial:ti,ab,kw)
AND
(reconstruct*:ti,ab,kw OR defect*:ti,ab,kw OR continuity:ti,ab,kw OR segmental:ti,ab,kw OR mandibulectom*:ti,ab,kw OR hemimandibulectom*:ti,ab,kw)
AND
('titanium'/exp OR titanium:ti,ab,kw OR timesh:ti,ab,kw OR 'ti mesh':ti,ab,kw)
AND
(mesh:ti,ab,kw OR meshes:ti,ab,kw OR scaffold*:ti,ab,kw OR cage*:ti,ab,kw OR tray:ti,ab,kw OR trays:ti,ab,kw OR grid:ti,ab,kw OR grids:ti,ab,kw OR 'titanium mesh':ti,ab,kw OR 'titanium mesh tray':ti,ab,kw OR 'titanium scaffold':ti,ab,kw OR 'titanium scaffolds':ti,ab,kw OR 'titanium cage':ti,ab,kw OR 'titanium cages':ti,ab,kw OR 'titanium tray':ti,ab,kw OR 'titanium trays':ti,ab,kw OR 'mesh tray':ti,ab,kw OR 'mesh trays':ti,ab,kw OR 'titanium grid':ti,ab,kw OR 'porous titanium':ti,ab,kw OR

'lattice titanium':ti,ab,kw)
AND
('patient-specific':ti,ab,kw OR 'patient specific':ti,ab,kw OR customized:ti,ab,kw OR customised:ti,ab,kw OR customize:ti,ab,kw OR customise:ti,ab,kw OR 'custom-made':ti,ab,kw OR 'custom made':ti,ab,kw OR personalized:ti,ab,kw OR personalised:ti,ab,kw OR 'cad/cam':ti,ab,kw OR 'cad-cam':ti,ab,kw OR 'computer-aided design':ti,ab,kw OR 'computer aided design':ti,ab,kw OR 'computer-assisted':ti,ab,kw OR 'computer assisted':ti,ab,kw OR 'virtual surgical planning':ti,ab,kw OR '3d printing':ti,ab,kw OR '3d printed':ti,ab,kw OR '3d-printed':ti,ab,kw OR 'three-dimensional printing':ti,ab,kw OR 'three-dimensional printed':ti,ab,kw OR 'additive manufacturing':ti,ab,kw OR 'additively manufactured':ti,ab,kw OR 'selective laser melting':ti,ab,kw OR slm:ti,ab,kw OR 'electron beam melting':ti,ab,kw OR ebm:ti,ab,kw OR 'direct metal laser sintering':ti,ab,kw OR dmls:ti,ab,kw OR pcbm:ti,ab,kw OR 'particulate cancellous bone':ti,ab,kw OR 'bone marrow':ti,ab,kw OR 'bone graft':ti,ab,kw OR 'bone grafts':ti,ab,kw OR 'bone grafting':ti,ab,kw OR 'bone regeneration':ti,ab,kw OR 'bone bridging':ti,ab,kw)

Database 3. Web of Science Core Collection

Records identified: 412

TS=(
(mandib* OR mandible OR jaw OR maxillofacial)
AND
(reconstruct* OR defect* OR continuity OR segmental OR mandibulectom* OR hemimandibulectom*)
AND
(titanium OR TiMesh OR "Ti mesh")
AND
(mesh OR meshes OR scaffold* OR cage* OR tray OR trays OR grid OR grids OR "titanium mesh" OR "titanium mesh tray" OR "titanium scaffold" OR "titanium scaffolds" OR "titanium cage" OR "titanium cages" OR "titanium tray" OR "titanium trays" OR "mesh tray" OR "mesh trays" OR "titanium grid" OR "porous titanium" OR "lattice titanium")
AND
("patient-specific" OR "patient specific" OR customized OR customised OR customize OR customise OR "custom-made" OR "custom made" OR personalized OR personalised OR "CAD/CAM" OR "CAD-CAM" OR "computer-aided design" OR "computer aided design" OR "computer-assisted" OR "computer assisted" OR "virtual surgical planning" OR "3D printing" OR "3D printed" OR "3D-printed" OR "three-dimensional printing" OR "three-dimensional printed" OR "additive manufacturing" OR "additively manufactured" OR "selective laser melting" OR SLM OR "electron beam melting" OR EBM OR "direct metal laser sintering" OR DMLS OR PCBM OR "particulate cancellous bone" OR "bone marrow" OR "bone graft" OR "bone grafts" OR "bone grafting" OR "bone regeneration" OR "bone bridging")
)

Database 4. Scopus

Records identified: 490

TITLE-ABS-KEY (
(mandib* OR mandible OR jaw OR maxillofacial)
AND
(reconstruct* OR defect* OR continuity OR segmental OR mandibulectom* OR hemimandibulectom*)
AND

(titanium OR TiMesh OR "Ti mesh")
AND
(mesh OR meshes OR scaffold* OR cage* OR tray OR trays OR grid OR grids OR "titanium mesh" OR "titanium mesh tray" OR "titanium scaffold" OR "titanium scaffolds" OR "titanium cage" OR "titanium cages" OR "titanium tray" OR "titanium trays" OR "mesh tray" OR "mesh trays" OR "titanium grid" OR "porous titanium" OR "lattice titanium")
AND
("patient-specific" OR "patient specific" OR customized OR customised OR customize OR customise OR "custom-made" OR "custom made" OR personalized OR personalised OR "CAD/CAM" OR "CAD-CAM" OR "computer-aided design" OR "computer aided design" OR "computer-assisted" OR "computer assisted" OR "virtual surgical planning" OR "3D printing" OR "3D printed" OR "3D-printed" OR "three-dimensional printing" OR "three-dimensional printed" OR "additive manufacturing" OR "additively manufactured" OR "selective laser melting" OR SLM OR "electron beam melting" OR EBM OR "direct metal laser sintering" OR DMLS OR PCBM OR "particulate cancellous bone" OR "bone marrow" OR "bone graft" OR "bone grafts" OR "bone grafting" OR "bone regeneration" OR "bone bridging")
)

Database 5. Cochrane Central Register of Controlled Trials (CENTRAL)

Records identified: 29

#1 mandib* OR mandible OR jaw OR maxillofacial:ti,ab,kw

#2 reconstruct* OR defect* OR continuity OR segmental OR mandibulectom* OR hemimandibulectom*:ti,ab,kw

#3 titanium OR TiMesh OR "Ti mesh":ti,ab,kw

#4 mesh OR meshes OR scaffold* OR cage* OR tray OR trays OR grid OR grids OR "titanium mesh" OR "titanium mesh tray" OR "titanium scaffold" OR "titanium scaffolds" OR "titanium cage" OR "titanium cages" OR "titanium tray" OR "titanium trays" OR "mesh tray" OR "mesh trays" OR "titanium grid" OR "porous titanium" OR "lattice titanium":ti,ab,kw

#5 "patient specific" OR customized OR customised OR customize OR customise OR "custom made" OR personalized OR personalised OR "CAD CAM" OR "computer aided design" OR "computer assisted" OR "virtual surgical planning" OR "3D printing" OR "3D printed" OR "three dimensional printing" OR "three dimensional printed" OR "additive manufacturing" OR "additively manufactured" OR "selective laser melting" OR SLM OR "electron beam melting" OR EBM OR "direct metal laser sintering" OR DMLS OR PCBM OR "particulate cancellous bone" OR "bone marrow" OR "bone graft" OR "bone grafts" OR "bone grafting" OR "bone regeneration" OR "bone bridging":ti,ab,kw

#6 #1 AND #2 AND #3 AND #4 AND #5

Database 6. ClinicalTrials.gov

Records identified: 11

(mandibular OR mandible OR jaw OR maxillofacial OR mandibulectomy OR hemimandibulectomy)
AND

(reconstruction OR reconstructive OR defect OR defects OR continuity OR segmental)
AND
(titanium OR "titanium mesh" OR "titanium scaffold" OR "titanium cage" OR "titanium tray" OR "mesh tray" OR "titanium grid" OR "porous titanium" OR "lattice titanium")
AND
("patient-specific" OR "patient specific" OR customized OR customised OR "custom-made" OR "custom made" OR personalized OR personalised OR "CAD/CAM" OR "CAD-CAM" OR "computer-aided design" OR "computer aided design" OR "computer-assisted" OR "computer assisted" OR "virtual surgical planning" OR "3D printing" OR "3D printed" OR "3D-printed" OR "three-dimensional printing" OR "three-dimensional printed" OR "additive manufacturing" OR "additively manufactured" OR "selective laser melting" OR "electron beam melting" OR "direct metal laser sintering" OR PCBM OR "particulate cancellous bone" OR "bone marrow" OR "bone graft" OR "bone grafting" OR "bone regeneration" OR "bone bridging")

Summary of records identified by source

Source	Records identified
PubMed	250
Embase	337
Web of Science Core Collection	412
Scopus	490
Cochrane Central Register of Controlled Trials (CENTRAL)	29
ClinicalTrials.gov	11
Total	1,529

Supplementary Table S2. Detailed characteristics of the 10 representative independent cohorts.

Author, year	Country	Institution	Study design	Recruitment	Total n	Eligible n	Denom. unit	Indication / etiology	Defect type	Device type	Manufacturing	Graft / adjuncts	RT	Follow-up	Notes / cohort-adjudication comments
Yamada 2016	Japan	Tsurumi University	Retrospective case series	2010.01-2014.06	21	19	patient	Benign 11 (ameloblastoma 8, KCOT 2, cementifying fibroma 1) + fibrous dysplasia/osteomyelitis 1 + malignant 9 (gingival SCC)	Segmental (L 14, LCL 2, C/LC/HC); 2 marginal excluded from eligible n	Custom titanium mesh tray (single-, then double-layer; laser-welded)	Non-AM (custom; CAD/3D-model pre-bent; laser-welded)	PCBM, posterior ilia +/- tibia	2 (30 Gy)	Mean 29 mo (9-62)	VSP + 3D skull model. Marginal cases (patients 3, 11) excluded from eligible n.
Onodera 2023	Japan	Iwate Medical University	Retrospective case series	2017.01.01-2021.12.31	18	12	patient	Benign 8 (ameloblastoma 5, myxoma 1, osteoblastoma 1, ossifying fibroma 1) + malignant 10 (gingival SCC 7, floor-of-mouth SCC 2,	Segmental 12 (Brown I/II/III) + marginal 6; none with condyle	Custom titanium mesh, pre-bent on 3D model (Leibinger Dynamic Mesh 0.6 mm;	Non-AM (custom; pre-bent on 3D-printed model)	PCBM, posterior iliac crest	None	Short-term; exact follow-up duration not extractable	VSP + 3D-printed model. Marginal cases (n=6) excluded from eligible n. Retained in the systematic review

Author, year	Country	Institution	Study design	Recruitment	Total n	Eligible n	Denom. unit	Indication / etiology	Defect type	Device type	Manufacturing	Graft / adjuncts	RT	Follow-up	Notes / cohort-adjudication comments
								mucoepidermoid 1)		double-layer when needed) + cutting guides					and sensitivity analyses but excluded from primary pooled estimates because a confirmable six-month follow-up was not reported.
Danjo 2025	Japan	Saga University	Retrospective case series (long-term)	1989.04-2016.03	21	21	patient	Malignant 10 + benign tumour/osteomyelitis 11	Segmental (CAT distribution: body 7, A 4, T 3, AT 4, TT 3); mean length 56.4 mm	Preformed titanium mesh tray (Dumbach 14 + OsteoMed shee	Non-AM (preformed mesh; 3D-model-assisted shaping)	Autogenous iliac: PCBM alone 14, block + PCBM 4, block only 3	7 irradiated	Post-prosthetic observation: mean 150.7 mo (ra	Follow-up is observation after implant-supported prosthesis delivery (post-loadin

Author, year	Country	Institution	Study design	Recruitment	Total n	Eligible n	Denom. unit	Indication / etiology	Defect type	Device type	Manufacturing	Graft / adjuncts	RT	Follow-up	Notes / cohort-adjudication comments
										t 7; laser-welded; shaped on 3D stereolithographic model)		(PCBM-predominant)		nge 4 mo-27 y 11 mo)	g), not time since reconstruction; implants were placed 4 mo-1 y after reconstruction, so reconstruction-level follow-up exceeds six months in all patients. Cohort selected to patients reaching implant prosthetic loading.

Author, year	Country	Institution	Study design	Recruitment	Total n	Eligible n	Denom. unit	Indication / etiology	Defect type	Device type	Manufacturing	Graft / adjuncts	RT	Follow-up	Notes / cohort-adjudication comments
															Long-term cohort.
Miyamoto 2014	Japan	Kyushu Dental University	Retrospective case series	2000-2011	9 patients / 10 sites	10 sites	site	Malignant SCC 6 + benign 3 (cemento-ossifying fibroma 2, ameloblastoma 1)	Segmental (body/ramus/chin/condyle); mean defect 62.4 mm	Titanium mesh tray (Dumbach / Dynamic Mesh / micromesh)	Non-AM (preformed/stock; intra-operatively bent)	PCBM, anterior/posterior iliac	3 (20/30/50 Gy)	Mean 83.2 mo (18-120)	Nine patients across 10 reconstructed sites; device-complication outcomes used site-level denominators. Outcome-specific denominators reported in Table 2 and Figure 2.
Iino 2009	Japan	University of	Multi-institut	1999-2004	15	15	patient	Malignant 11 + osteomyelitis 2	Segmental (body +/-	Dumbach Titan	Non-AM (preform	PCBM, bilateral	6 (40-50	Mean 62.7	Independent earlier multi-

Author, year	Country	Institution	Study design	Recruitment	Total n	Eligible n	Denom. unit	Indication / etiology	Defect type	Device type	Manufacturing	Graft / adjuncts	RT	Follow-up	Notes / cohort-adjudication comments
(Dumbach)		Tokyo / Akita / Tsurumi (multi-institution)	ion retrospective case series					+ ameloblastoma 1 + trauma 1	chin/ramus)	Mesh-System (ready-made tray)	ed/stock; ready-made)	posterior ilia; PRP in 10/15	Gy)	mo (29-108)	institution Dumbach cohort; recruitment 1999-2004 and ready-made device era distinct from the later Tsurumi cohort (Yamada 2016, 2010-2014); counted once. Reoperation/device removal not separately extractable for the primary

Author, year	Country	Institution	Study design	Recruitment	Total n	Eligible n	Denom. unit	Indication / etiology	Defect type	Device type	Manufacturing	Graft / adjuncts	RT	Follow-up	Notes / cohort-adjudication comments
															combined outcome; outcome-specific decisions in Table 2 and Figure 2.
Ferretti 2002	South Africa	University of the Witwatersrand	Prospective alternate-allocation comparative study	NR	13	13	patient	Gunshot/trauma 6 + ameloblastoma 5 + ossifying fibroma 1 + desmoplastic fibroma 1	Segmental continuity defects up to ~6+ cm	Preformed titanium mesh tray (Howmedica-Leibinger)	Non-AM (preformed/stock)	Corticocancellous block (ABG arm) +/- bovine BMP/OP on human DBM (osteogenic-device arm); no PCB	NR	13 mo-4 y (2 lost at 5-6 mo)	Prospective alternate-allocation comparative study; ABG (n=7) vs osteogenic device (n=6); same preformed tray in both arms. Assessed with ROBINS-I

Author, year	Country	Institution	Study design	Recruitment	Total n	Eligible n	Denom. unit	Indication / etiology	Defect type	Device type	Manufacturing	Graft / adjuncts	RT	Follow-up	Notes / cohort-adjudication comments
												M reported			because allocation was alternate rather than truly randomized. Contextual/exploratory; not in primary pooled estimates.
Shehab 2018	Egypt	Cairo University	Prospective case series	2014.03-2015.06	8	8	patient	Benign (follicular ameloblastoma/ameloblastoma 6, KCOT 2)	Segmental; 2 cases crossed midline / >9 cm	Patient-specific titanium mesh tray (two fixation arms)	AM-additively manufactured (electron beam melting, EBM)	Particulate iliac bone + marrow (PCBM-equivalent) + PRF	None (excluded)	1 y, as reported	CAD (Mimics) + mirror imaging; titanium tray EBM-printed (Arcam). PRF adjunct membrane.
Zh	C	Fou	Pro	NR	6	6	p	Benign	Discont	Cust	Non	Parti	N	Me	Metal

Author, year	Country	Institution	Study design	Recruitment	Total n	Eligible n	Denom. unit	Indication / etiology	Defect type	Device type	Manufacturing	Graft / adjuncts	RT	Follow-up	Notes / cohort-adjudication comments
ou 2010	hina	rth Military Medical University, Xi'an	spective case series				atient	5 (ameloblastoma, KCOT, neurofibroma, dentigerous cyst) + osteomyelitis 1	inuous (body/ramus/symphysis; some condyle)	omized cast titanium tray (CAD-designed; 0.8 mm wall; rigid)	-AM (customized cast; resin model followed by investment casting)	culate cancellous iliac bone + marrow (PCBM-equivalent) + cortical blocks	R	an 50 mo (39-68)	device cast (not printed); reverse-engineering/CAD/rapid-prototyping; stereolithography resin model only.
Zhao 2023	China	Shanghai Ninth People's Hospital	Prospective case series	NR	7	7	patient	Recurrent or large mandibular ameloblastoma 7	Segmental (body/molar/ramus)	Custom 3D-printed titanium mesh (porous; selective laser melting)	AM - additively manufactured (selective laser melting, SLM)	Cancellous bone, posterior iliac (marrow not specified)	NR	6 mo	CAD (ProPlan CMF) + mirror imaging; mesh SLM-printed (Concept Laser M2); marrow not stated in source.

Author, year	Country	Institution	Study design	Recruitment	Total n	Eligible n	Denom. unit	Indication / etiology	Defect type	Device type	Manufacturing	Graft / adjuncts	RT	Follow-up	Notes / cohort-adjudication comments
Sato 2022	Japan	Showa University	Retrospective case series	2014-2020	5	5	patient	Ameloblastoma 5	Segmental; defect 5-12 cm	Custom titanium mesh cage 0.6 mm (Ultra Flex Mesh Custom; hexagonal)	Non-AM (custom; pre-bent on 3D-printed model)	PCBM, anterior iliac	NR	16-88 mo (mean 44.6)	Navigation-assisted; 3D-printed model; cage pre-bent in laboratory. Follow-up and graft details extracted as reported.

Manufacturing approach (strict): AM = Shehab 2018 (EBM) and Zhao 2023 (SLM) only; all other cohorts are non-AM (Zhou 2010 customized cast; Yamada/Onodera/Sato custom model-bent; Danjo/Miyamoto/Iino/Ferretti preformed or stock).

Onodera 2023 was retained in the systematic review and sensitivity analyses but excluded from the primary pooled estimates because a confirmable six-month follow-up was not reported.

Danjo 2025 follow-up is observation after implant-supported prosthesis delivery (post-prosthetic loading), not time since reconstruction; reconstruction-level follow-up exceeds six months in all patients.

Miyamoto 2014 comprised 9 patients across 10 reconstructed sites; primary device-complication denominators were site-level.

Keller 1998, Herford 2008 and Tokyo Medical (Matsuo 2007 / Iwamoto 2009) are not representative cohorts and are not listed here.

Abbreviations: ABG, autogenous bone graft; AM, additively manufactured; BMP, bone morphogenetic protein; CAD, computer-aided design; DBM, demineralized bone matrix; EBM, electron beam melting; Gy, gray; KCOT, keratocystic odontogenic tumour; mo, months; NR, not reported; OP, osteogenic protein; PCBM, particulate cancellous bone and marrow; PRF, platelet-rich fibrin; PRP, platelet-rich plasma; ROBINS-I, Risk Of Bias In Non-randomised Studies of Interventions; RT, radiotherapy; SCC, squamous cell carcinoma; SLM, selective laser melting; VSP, virtual surgical planning; y, years.

Supplementary Table S3. Sensitivity analyses for the four primary device-related outcomes.

Outcome	Analysis	Cohorts, k	Events / denominator	Pooled, %	95% CI, %	I2, %	tau2	Notes
Device exposure	Primary GLMM, excluding Onodera 2023	5	5/57	8.8	3.7-19.4	0	0	Primary analysis
	Sensitivity GLMM, including Onodera 2023	6	7/69	10.1	4.9-19.8	0	0	Influence of Onodera 2023
	Freeman-Tukey random-effects model	5	5/57	7.0	0.8-16.8	0	—	Model-based check
	Fixed-effect model	5	5/57	11.0	4.8-23.2	—	—	Reference only
	Leave-one-out (range)	5	5/57	—	6.1-10.5	—	—	Range across single-cohort omissions
Infection	Primary GLMM, excluding Onodera 2023	7	12/83	14.5	8.4-23.8	0	0	Primary analysis
	Sensitivity GLMM, including Onodera 2023	8	14/95	14.7	8.9-23.4	0	0	Influence of Onodera 2023
	Freeman-Tukey random-effects model	7	12/83	12.5	5.2-21.7	0	—	Model-based check
	Fixed-effect model	7	12/83	16.1	9.4-26.3	—	—	Reference only
	Leave-one-out (range)	7	12/83	—	12.8-16.1	—	—	Range across single-cohort omissions
Mechanical failure	Primary GLMM,	7	4/76	5.3	2.0-13.2	0	0	Primary analysis

Outcome	Analysis	Cohorts, k	Events / denominator	Pooled, %	95% CI, %	I2, %	tau2	Notes
	excluding Onodera 2023							
	Sensitivity GLMM, including Onodera 2023	8	4/88	4.5	1.7-11.5	0	0	Influence of Onodera 2023
	Freeman-Tukey random-effects model	7	4/76	2.8	0.0-9.6	0	—	Model-based check
	Fixed-effect model	7	4/76	8.3	3.8-17.3	—	—	Reference only
	Leave-one-out (range)	7	4/76	—	3.5-6.1	—	—	Range across single-cohort omissions
Unplanned reoperation or device removal	Primary GLMM, excluding Onodera 2023	6	10/69	14.5	8.0-24.9	0	0	Primary analysis
	Sensitivity GLMM, including Onodera 2023	7	12/81	14.8	8.6-24.3	0	0	Influence of Onodera 2023
	Freeman-Tukey random-effects model	6	10/69	13.3	5.1-23.6	0	—	Model-based check
	Fixed-effect model	6	10/69	15.1	8.3-25.9	—	—	Reference only
	Leave-one-out (range)	6	10/69	—	13.1-16.0	—	—	Range across single-cohort omissions

Primary analyses excluded Onodera 2023 because a confirmable minimum six-month follow-up duration was not reported. Sensitivity analyses including Onodera 2023 were performed to assess the influence of this cohort. Freeman–Tukey double-arcsine and fixed-effect models were used

as model-based sensitivity checks. Leave-one-out analyses were performed when at least five cohorts contributed to an outcome; values represent the range of pooled proportions across single-cohort omissions. $I^2 = 0\%$ and $\tau^2 = 0$ indicate that between-study heterogeneity could not be reliably estimated under small numbers of cohorts and sparse events, rather than evidence of true homogeneity. Abbreviations: CI, confidence interval; GLMM, generalized linear mixed model.

Supplementary Table S4. Risk-of-bias assessments.

A. JBI Critical Appraisal Checklist for Case Series

Study	Consecutive inclusion	Complete inclusion	Demographics / clinical info	Intervention / exposure	Outcome measurement	Follow-up	Statistical analysis	Overall judgement	Main concerns
Yamada 2016	Unclear	Unclear	Yes	Yes	Yes	Yes	Yes	Moderate concerns	Consecutive/complete inclusion not stated (retrospective).
Onodera 2023	Yes	Yes	Yes	Yes	Yes	Unclear	Yes	Low to moderate concerns	Follow-up duration not extractable.
Danjo 2025	Unclear	No	Yes	Yes	Yes	Unclear	Unclear	Moderate to high concerns	Incomplete inclusion (cohort selected to implant loading); limited reporting of some denominators/ analysis.
Miyamoto 2014	Unclear	Unclear	Yes	Yes	Yes	Yes	Yes	Moderate concerns	Consecutive/complete inclusion not stated.
Iino 2009 (Dumbach)	Unclear	Unclear	Yes	Yes	Yes	Yes	Yes	Moderate concerns	Consecutive/complete inclusion not stated (multi-institution).
Shehab 2018	Yes	Yes	Yes	Yes	Yes	Yes	Yes	Low concerns	Small sample; otherwise well reported.
Zhou 2010	Unclear	Unclear	Yes	Yes	Yes	Yes	Unclear	Moderate concerns	Small sample; descriptive analysis only.
Zhao 2023	Yes	Yes	Yes	Yes	Yes	Unclear	Yes	Low to moderate concerns	Short follow-up; coarse complication reporting.

Study	Consecutive inclusion	Complete inclusion	Demographics / clinical info	Intervention / exposure	Outcome measurement	Follow-up	Statistical analysis	Overall judgement	Main concerns
Sato 2022	Unclear	Unclear	Yes	Yes	Yes	Yes	Unclear	Moderate concerns	Small sample; limited statistical reporting.
Keller 1998	Unclear	Unclear	Yes	Yes	Yes	Yes	Yes	Moderate concerns	Eligible titanium-mesh subgroup implant-selected and not separable; qualitative only.
Herford 2008	Unclear	Unclear	Unclear	Yes	Yes	Yes	Unclear	Moderate to high concerns	Incomplete per-case reporting; mesh subgroup not separable; qualitative only.
Matsuo 2007 / Iwamoto 2009 (Tokyo Medical)	Unclear	Unclear	Partly	Yes	Yes	Yes	Unclear	Moderate to high concerns	Defect type not tabulated; small mixed cohort; qualitative only.

B. ROBINS-I (prospective alternate-allocation comparative study)

Study	Confounding	Selection of participants	Classification of interventions	Deviations	Missing data	Measurement of outcomes	Selection of reported result	Overall judgement	Main concerns
Ferretti 2002 (prospective alternate-allocation compar	Moderate	Moderate	Low	Low	Moderate	Moderate	Moderate	Moderate concerns	Non-random (alternate) allocation; small sample; 2 of 13

Study	Confounding	Selection of participants	Classification of interventions	Deviations	Missing data	Measurement of outcomes	Selection of reported result	Overall judgement	Main concerns
ative study)									lost to follow-up at 5-6 months. Assessed with ROBINS-I because allocation was alternate rather than truly randomized.

C. JBI Critical Appraisal Checklist for Case Reports (contextual evidence map)

Report	Demographics	History / timeline	Clinical condition	Diagnostic assessment	Intervention	Post-intervention outcome	Adverse events	Overall judgement	Notes
Malekpour 2014 (evidence map)	Yes	Yes	Yes	Yes	Yes	Yes	Unclear	Low to moderate concerns	Single case (contextual evidence map); adverse events not systematically reported. Other evidence-map single-case/technical reports were not individually

Report	Demographics	History / timeline	Clinical condition	Diagnostic assessment	Intervention	Post-intervention outcome	Adverse events	Overall judgement	Notes
									appraised and contribute to no pooled estimate.

Risk of bias was assessed using the Joanna Briggs Institute checklist for case series or case reports, as applicable, and ROBINS-I for the prospective alternate-allocation comparative study. Ferretti 2002 was assessed with ROBINS-I because allocation was alternate rather than truly randomized. Judgements were used to inform interpretation and certainty of evidence; studies were not excluded solely on the basis of methodological quality. Abbreviations: JBI, Joanna Briggs Institute; ROBINS-I, Risk Of Bias In Non-randomized Studies - of Interventions.

Supplementary Table S5. GRADE evidence profile for the four primary device-related outcomes.

Outcome	Study design / evidence type	Cohorts, k	Events / denominator	Pooled proportion, %	95% CI, %	Risk of bias	Inconsistency	Indirectness
Device exposure	Single-arm observational evidence	5	5/57	8.8	3.7-19.4	Serious (single-arm case-series design).	Not downgraded; heterogeneity could not be reliably estimated because of small numbers of cohorts and sparse events.	Not serious.
Infection	Single-arm observational evidence	7	12/83	14.5	8.4-23.8	Serious (single-arm case-series design).	Not downgraded; heterogeneity could not be reliably estimated because of small numbers of cohorts and sparse events.	Not serious.
Mechanical failure	Single-arm observational evidence	7	4/76	5.3	2.0-13.2	Serious (single-arm case-series design).	Not downgraded; heterogeneity could not be reliably estimated because of small numbers of cohorts and sparse events.	Not serious.

Outcome	Study design / evidence type	Cohorts, k	Events / denominator	Pooled proportion, %	95% CI, %	Risk of bias	Inconsistency	Indirectness
Unplanned reoperation or device removal	Single-arm observational evidence	6	10/69	14.5	8.0-24.9	Serious (single-arm case-series design).	Not downgraded; heterogeneity could not be reliably estimated because of small numbers of cohorts and sparse events.	Not serious.

Certainty of evidence was assessed using GRADE. Because all outcomes were based on single-arm observational proportions from small clinical series, the certainty of evidence was rated as very low. I2 = 0% and tau2 = 0 were not interpreted as evidence of true homogeneity because heterogeneity could not be reliably estimated under small numbers of cohorts and sparse events. Abbreviations: CI, confidence interval; GRADE, Grading of Recommendations Assessment, Development and Evaluation.
